# Tridecaptin M, a New Variant Discovered in Mud Bacterium, Shows Activity against Colistin- and Extremely Drug-Resistant *Enterobacteriaceae*

**DOI:** 10.1128/AAC.00338-19

**Published:** 2019-05-23

**Authors:** Manoj Jangra, Manpreet Kaur, Rushikesh Tambat, Rohit Rana, Sushil K. Maurya, Neeraj Khatri, Abdul Ghafur, Hemraj Nandanwar

**Affiliations:** a Clinical Microbiology and Bioactive Screening Laboratory, CSIR-Institute of Microbial Technology, Chandigarh, India; b Natural Product Chemistry and Process Development Division, CSIR-Institute of Himalayan Bioresource Technology, Palampur, Himachal Pradesh, India; c Animal House Facility, CSIR-Institute of Microbial Technology, Chandigarh, India; d Infectious Diseases, Apollo Specialty Hospitals, Chennai, Tamilnadu, India; e Academy of Scientific and Innovative Research, CSIR-HRDC, Ghaziabad, Uttar Pradesh, India

**Keywords:** Gram-negative bacteria, *Paenibacillus* sp., XDR *Enterobacteriaceae*, antibacterial drug screening, antibiotic resistance, antimicrobial agents, colistin resistance, nonribosomal peptides, tridecaptin M

## Abstract

The World Health Organization has categorized the Gram-negative superbugs, which are inherently impervious to many antibiotics, as critical priority pathogens due to the lack of effective treatments. The breach in our last-resort antibiotic (i.e., colistin) by extensively drug-resistant and pan-drug-resistant *Enterobacteriaceae* strains demands the immediate development of new therapies. In the present study, we report the discovery of tridecaptin M, a new addition to the family, and its potential against colistin-resistant *Enterobacteriaceae in vitro* and *in vivo*. Also, we performed mode-of-action studies using various fluorescent probes and studied the hemolytic activity and mammalian cytotoxicity in two cell lines. Tridecaptin M displayed strong antibacterial activity (MICs of 2 to 8 μg ml^−1^) against clinical strains of Klebsiella pneumoniae (which were resistant to colistin, carbapenems, third- and fourth-generation cephalosporins, fluoroquinolones, fosfomycin, and other antibiotics) and *mcr-1*-positive Escherichia coli strains. Unlike polymyxins, tridecaptin M did not permeabilize the outer membrane or cytoplasmic membrane. It blocked ATP synthesis in bacteria by dissipating the proton motive force. The compound exhibited negligible acquired resistance, low *in vitro* cytotoxicity and hemolytic activity, and no significant acute toxicity in mice. It also showed promising efficacy in a thigh infection model of colistin-resistant K. pneumoniae. Altogether, these results demonstrate the future prospects of this class of antibiotics to address the unmet medical need to circumvent colistin resistance in extensively drug-resistant *Enterobacteriaceae* infections. The work also emphasizes the importance of natural products in our shrunken drug discovery pipeline.

## Abstract (precis)

The World Health Organization has categorized the Gram-negative superbugs, which are inherently impervious to many antibiotics, as critical priority pathogens due to the lack of effective treatments. The breach in our last-resort antibiotic (i.e., colistin) by extensively drug-resistant and pan-drug-resistant *Enterobacteriaceae* strains demands the immediate development of new therapies.

## INTRODUCTION

The discovery of penicillin in 1928 transformed the world of medical science and saved millions of lives. This was followed by a “golden era” of antibiotics, during which many new classes were uncovered from natural sources (1). Bacteria developed a counter-mechanism, however, during evolution, to neutralize the effects of antibiotics to which they were exposed. Treating common infections with global capacity relies on the constant supply of effective antibiotics. In comparing the rate of spread of drug resistance with the numbers of new antimicrobials approved, the number has decreased to only 9 antibiotics in the past decade, from 29 in the 1980s and 23 in the 1990s. Recently, some innovative contributions have added to our antibiotic arsenal, e.g., teixobactin, lugdunin, pseudouridimycin, and malacidins (2–5). Notably, all of these newly isolated classes of antibiotics exert their effects in Gram-positive bacteria only. According to the Pew Charitable Trusts analysis of the antibiotic pipeline (6), only 11 antibiotics in clinical development could address infections caused by critical pathogens mentioned in the WHO priority list (7).

Extensively drug-resistant (XDR) and pan-drug-resistant (PDR) strains of *Enterobacteriaceae* cause severe nosocomial and community infections, which are now becoming untreatable due to the scarcity of effective antibiotics (8–10). In the early 2000s, carbapenem-resistant *Enterobacteriaceae* strains forced clinicians to include in their drug regimens a previously discarded antibiotic, polymyxin E (or colistin), as an antibiotic of last resort (11). Colistin is a cationic lipodecapeptide that exerts its bactericidal effect through initial electrostatic interactions with lipopolysaccharide (LPS), membrane permeabilization leading to cell content leakage, and eventually cell death (12). However, massive use of this important antibiotic in agriculture and poultry has increased the resistance in clinical pathogens (13). Colistin resistance is attributed to alteration of the lipid A biosynthetic pathway and modification of LPS (14–18). This breach in our last line of antibiotic defense by deadly pathogens is a serious public health issue. Colistin resistance has alarmed the scientific community and pharmaceutical companies to develop new weapons against XDR and PDR strains (19–21). Smith et al. recently reported a new class of antibiotics, arylomycins, which are effective against ESKAPE (Enterococcus faecium, Staphylococcus aureus, Klebsiella pneumoniae, Acinetobacter baumannii, Pseudomonas aeruginosa, and *Enterobacter* species) pathogens (22). A few more antibiotics have been developed or in the development stage, such as POL7080 and plazomycin (6), but they are all prone to the development of resistance. Therefore, given the lesser chances of approval of a newly discovered antibiotic for final use and the profound crisis in available therapies against Gram-negative pathogens, we must tackle this global challenge by continuously screening for new antibiotics.

Tridecaptins, which were initially discovered in 1978, are a group of nonribosomally synthesized lipopeptide antibiotics that retain activity against Gram-negative bacteria (23–26). They possess a different mechanism of action than polymyxins and kill Gram-negative bacteria by binding to lipid II and causing membrane disruption (25). Although these antibiotics show promising activity against Gram-negative pathogens, very few studies have been carried out to determine their potential in the shrunken drug discovery pipeline. Furthermore, no reports on whether these antibiotics would work against colistin-resistant superbugs are available. Here, we show the discovery of a new variant of this family, tridecaptin M, from a mud bacterium, which intrigued us to study its antibacterial potential against colistin-resistant and XDR K. pneumoniae isolates *in vitro* and *in vivo*.

## RESULTS

### Isolation and structural characterization of tridecaptin M.

The isolate M-152 inhibited both Gram-positive and Gram-negative bacteria and was identified as *Paenibacillus* sp. based on 16S rRNA gene sequencing (99.5% similarity with Paenibacillus jamilae) and matrix-assisted laser desorption ionization–time of flight mass spectrometry (MALDI-TOF MS) analysis. Our group was also involved in a parallel study of polymyxin A from Paenibacillus dendritiformis (27). We hoped that polymyxin A would demonstrate activity toward colistin-resistant strains, because insufficient data were available on cross-resistance among polymyxin variants. Unfortunately, however, the activity of polymyxin A did not differ from that of colistin (and polymyxin B) in those strains. This finding led us to conclude that a microbe that produced any polymyxin compound would most likely not inhibit bacteria resistant to the same or any other polymyxin variant. We set up an assay with polymyxin-sensitive and -resistant strains of K. pneumoniae using a crude fermentation extract of M152. The extract inhibited the two cultures at similar concentrations, with a difference of just 2- to 4-fold, whereas this difference appeared to be 32-fold for colistin. The clinical isolate (AH-16) used in the assay was resistant to almost every class of antibiotics (as described below). We hypothesized that this activity corresponded either to a new antibiotic class that shares no cross-resistance with polymyxins (or any other antibiotics) or to some kind of synergism. We proceeded to identify the active ingredient(s) responsible for this phenomenon.

**FIG 1:**
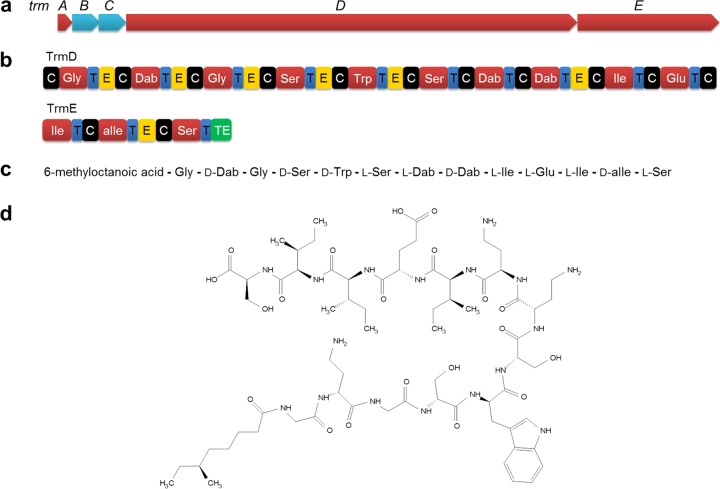
Predicted biosynthetic gene cluster of M152-P3 and the two-dimensional structure determined by MS and NMR spectroscopy. (a) Organization of biosynthetic genes for M152-P3. *trmB* and *trmC* are ABC transporter-encoding genes, *trmD* and *trmE* encode NRPSs, and *trmA* is a putative thioesterase gene. (b) Different modules of NRPSs. C, condensation domain; T, thiolation or peptide carrier domain; E, epimerase; TE, thioesterase. Adenylation domains are represented by the amino acid codes. (c) Linear peptide sequence of tridecaptin M encoded by NRPSs, also showing the position of d-amino acids. (d) Two-dimensional structure of tridecaptin M (or M152-P3).

Through bioactivity-guided fractionation with cation-exchange chromatography and reverse-phase high-performance liquid chromatography (HPLC), we isolated one compound (initially named M152-P3) from the crude extract. The signal of the compound appeared at *m/z* 1,488.8246 (M+H) in high-resolution mass spectrometry (HRMS) (see Fig. S1 in the supplemental material), suggesting the molecular ion as 1,487.8167. Liquid chromatography-electrospray ionization mass spectrometry (LC-ESI MS) revealed the monoisotopic mass of 1,487.8344 Da (Fig. S2). These data helped us to deduce the molecular formula, C_68_H_113_N_17_O_20_ (calculated monoisotopic mass, 1,487.8348). We then proceeded to solve the structure of this compound. The daughter ions generated in tandem mass spectrometry (MS/MS) analyses were assigned using Peaks Studio 8.5 (28). Unfortunately, the software could not predict some of the ions due to the presence of unusual amino acids; therefore, we manually curated the *de novo* sequence, assigned all of the b and y ions (Fig. S3), and further evaluated the sequence by amino acid analysis (Fig. S4). The data were consistent except that tryptophan was not detected in the amino acid analysis; however, the compound showed absorbance at 280 nm, which supported the presence of a tryptophan moiety in the compound. The amino acid composition and a tentative sequence derived from MS/MS analysis suggested that this compound belongs to the tridecaptin family (23, 26, 29). The MS/MS sequence was consistent with that of tridecaptin B at all positions except 1. At position 11, M152-P3 displayed isoleucine, while tridecaptin B has a valine at this place. Also, tridecaptin B possesses 6-methyloctanoic acid at the N terminus, and it was difficult to determine whether the residual mass of 158 Da obtained in MS/MS analysis of M152-P3 at the N terminus is 6-methyloctanoic acid or linear nonanoic acid. To solve this puzzle and to deduce the complete structural assignment of M152-P3, we performed one-dimensional and two-dimensional (heteronuclear single quantum correlation [HSQC], heteronuclear multiple bond correlation [HMBC], homonuclear correlation spectroscopy [COSY], and total correlation spectroscopy [TOCSY]) NMR spectroscopy. The NMR data removed all of the ambiguities that appeared in the initial stages. We could clearly observe the tryptophan signals in the NMR spectra, and the presence of isoleucine at position 11 was confirmed. NMR spectroscopy also revealed that the fatty acid moiety at the N terminus was 6-methyloctanoic acid, since a methyl group showing coupling to the methine carbon (at position 6) of the lipid tail was visible. The complete NMR assignment and the raw NMR spectra are provided in the supplemental material (Fig. S5 to S12 and Table S1). The stereochemistry of the amino acids was assigned based on Marfey’s analysis. The low biological yield (<1 mg liter^−1^) of M152-P3 in fermentation caused limitations in the analysis of the stereochemistry of the lipid tail, because we need a large amount of peptide to obtain enough fatty acid upon hydrolysis (less than 10% of the total compound) (26). The chiral methyl group present in the lipid chain of all natural tridecaptin variants is in the *S*-configuration because, in the *Bacillus* genus, it is mostly derived from l-isoleucine (26, 30). Therefore, we considered the methyl group as being in the *S*-configuration. Also, the stereochemistry of the methyl group does not contribute significantly to the activity (31). Figure 1d shows the two-dimensional structure of M152-P3. Considering the MS, amino acid analysis, and NMR data, we concluded that M152-P3 is a new variant of this family and differs from tridecaptin B at 1 amino acid position. We named this variant tridecaptin M (the letter M is derived from the village Malvi [29.0980°N, 76.3391°E], Jind, Haryana, India, from where the mud sample was collected).

### Biosynthetic gene cluster.

To confirm the production of tridecaptin M by the bacterium M-152, we analyzed the whole-genome of this strain. antiSMASH predicted a single tridecaptin biosynthetic gene cluster responsible for the synthesis of M152-P3 (Fig. 1a), which was homologous to the previously reported gene clusters of tridecaptin A (32) and tridecaptin B (26). The cluster consisted of 5 genes encoding different proteins for synthesis and secretion of active peptide, e.g., *trmA* (a putative thioesterase), *trmB* and *trmC* (ABC transporters), and *trmD* and *trmE* (nonribosomal peptide synthetases [NRPSs]). The two main biosynthetic enzyme complexes (TrmD and TrmE) together were composed of 13 adenylation domains, each responsible for incorporation of 1 amino acid (Fig. 1b). We could also observe the epimerase domains next to seven adenylation domains. These domains convert the stereochemistry of l-amino acids to the d-form (33, 34). The positions of the epimerase domains were consistent with the experimentally observed d-amino acids in the compound (Fig. 1c) and with other variants in this family. Of note, we observed some discrepancies in the specificities of some adenylation domains predicted by software programs (Table S2). Similar results were reported for gene clusters of tridecaptin A (32) and tridecaptin B (26). The biosynthetic gene cluster of M152-P3 differed from other tridecaptin gene clusters by 10% to 15%.

### Tridecaptin M activity in colistin-resistant Gram-negative bacteria.

We next sought to determine the biological activity of this variant in different pathogens. M152-P3 showed narrow-spectrum activity against Gram-negative bacteria (Table 1), with better MICs against the *Enterobacteriaceae* family. In Escherichia coli and K. pneumoniae strains, the MICs varied from 0.5 to 8 μg ml^−1^. The compound also displayed efficacy against multidrug-resistant (MDR) clinical isolates of K. pneumoniae, which were all polymyxin sensitive. Although the compound had 2- to 4-fold higher MICs than colistin and polymyxin B in polymyxin-sensitive strains, it exhibited a much better spectrum in polymyxin-resistant K. pneumoniae isolates (Table 2). Some of the clinical isolates displayed XDR profiles, but they were all sensitive to M152-P3 (MICs of 2 μg ml^−1^), unlike polymyxins, which had poor activity. M152-P3 was not active against Gram-positive bacteria or Candida albicans (no MIC was achieved up to 128 μg ml^−1^). The MICs were also determined in cation-adjusted Mueller-Hinton broth (CA-MHB) supplemented with 5% (vol/vol) fetal bovine serum and the MICs remained unchanged (data not shown), suggesting the stability of this compound.

**TABLE 1:** Antibacterial spectrum of M152-P3 in various pathogens

Strain	MIC (μg ml^−1^)^*a*^		
	M152-P3	Polymyxin B	Colistin
Klebsiella pneumoniae ATCC 700603	4	1	1
K. pneumoniae ATCC BAA-1705	2	1	1
K. pneumoniae ATCC BAA-1706	4	1	1
K. pneumoniae ATCC BAA-2146	2	1	1
K. pneumoniae ATCC 15380	1	1	1
K. pneumoniae ATCC 29665	0.5	1	1
K. pneumoniae subsp. *rhinoscleromatis* ATCC 13384	4	1	0.5
K. oxytoca MTCC 8295	2	ND	1
Enterobacter aerogenes MTCC 10208	4	ND	1
E. cloacae MTCC 509	4	ND	ND
Escherichia coli ATCC 25922	4	1	1
Escherichia coli ATCC 35218	4	ND	ND
Escherichia coli 9062 (clinical isolate)	4	1	1
Escherichia coli 7932 (clinical isolate)	4	1	1
Pseudomonas aeruginosa ATCC 27853	16	2	1
Acinetobacter baumannii ATCC 19606	>32	2	1
Salmonella enterica ATCC 10708	4	ND	1
K. pneumoniae MDR (polymyxin-sensitive) clinical isolates (19 clinical strains) (range)	2–8	1–2	0.5–2

a ND, not determined.

**TABLE 2:** Activity of M152-P3 in colistin-resistant and MDR *Enterobacteriaceae* strains

Species and strain	Resistance phenotype^*a*^	MIC (μg/ml)		
		M152-P3	Polymyxin B	Colistin
K. pneumoniae (clinical isolates)				
AH-1	G, CIP, CTR, MRP, AMP, TOB, FO, CAZ, NIT, AK, P/T, NX, A/S, AT, PIP, CTX, IMP	2	32	32
AH-2	G, CIP, CTR, MRP, AMP, TOB, FO, CAZ, NIT, AK, P/T, NX, A/S, AT, PIP, CTX, IMP	2	8	8
AH-3	G, CIP, CTR, MRP, AMP, TOB, FO, CAZ, NIT, AK, TE, P/T, NX, A/S, AT, PIP, CTX, IMP	2	4	8
AH-4	G, CIP, CTR, MRP, AMP, TOB, FO, CAZ, NIT, AK, P/T, NX, A/S, AT, PIP, CTX, IMP	2	4	8
AH-5	G, CIP, CTR, MRP, AMP, TOB, FO, CAZ, NIT, AK, P/T, NX, A/S, AT, PIP, CTX, IMP	2	8	8
AH-6	G, CIP, CTR, MRP, AMP, TOB, FO, CAZ, NIT, AK, P/T, NX, A/S, AT, PIP, CTX, IMP	2	4	4
AH-9	G, CIP, CTR, MRP, AMP, TOB, FO, CAZ, NIT, AK, P/T, NX, A/S, AT, PIP, CTX, IMP	2	4	8
AH-10	G, CIP, CTR, MRP, AMP, TOB, FO, CAZ, NIT, AK, P/T, NX, A/S, AT, PIP, CTX, IMP	2	16	16
AH-11	G, CIP, CTR, MRP, AMP, TOB, FO, CAZ, NIT, AK, P/T, NX, A/S, AT, PIP, CTX, IMP	2	16	16
AH-12	G(I), CIP, CTR, MRP, AMP, TOB, CAZ, NIT, AK, TE(I), P/T, NX, A/S, AT, PIP, CTX, IMP	2	16	32
AH-13	G, CIP, CTR, MRP, AMP, TOB, FO, CAZ, NIT, AK, P/T, NX, A/S, AT, PIP, CTX, IMP	2	4	8
AH-14	G, CIP, CTR, MRP, AMP, TOB, CAZ, NIT, AK, P/T, NX, A/S, AT, PIP, CTX, IMP	2	4	4
AH-15	G, CIP, CTR, MRP, AMP, TOB, FO, CAZ, NIT, AK, P/T, NX, A/S, AT, PIP, CTX, IMP	2	4	4
AH-16	G(I), CIP, CTR, MRP, AMP, TOB, FO, CAZ, NIT, AK, TE, P/T, NX, A/S(I), AT, PIP, CTX, IMP	2	32	32
AH-17	G, CIP, CTR, MRP, AMP, TOB, FO, CAZ, NIT, AK, P/T, NX, A/S, AT, PIP, CTX, IMP	2	32	32
AH-18	G, CIP, CTR, MRP, AMP, TOB, FO, CAZ, NIT, AK, P/T, NX, A/S, AT, PIP, CTX, IMP	2	32	32
AH-19	G, CIP, CTR, MRP, AMP, TOB, FO, CAZ, NIT, AK, P/T, NX, A/S, AT, PIP, CTX, IMP	2	32	32
E. coli (food isolates)^*b*^				
CF-23	*mcr* positive	4	ND	8
CF-45	*mcr* positive	4	ND	4
CF-47	*mcr* positive	4	ND	4

a G, gentamycin; CIP, ciprofloxacin; CTR, ceftriaxone; MRP, meropenem; AMP, ampicillin; TOB, tobramycin; FO, fosfomycin; CAZ, ceftazidime; NIT, nitrofurantoin; AK, amikacin; TE, tetracycline; P/T, piperacillin-tazobactam; NX, norfloxacin; A/S, ampicillin-sulbactam; AT, aztreonam; PIP, piperacillin; CTX, cefotaxime; IMP, imipenem. I in parentheses indicates intermediate resistance.

b These strains were isolated from food samples and are sensitive to most of the other antibiotics (62). They were included in the study because they are colistin resistant due to the presence of the *mcr-1* gene. The *Klebsiella* isolates have mutations in the *mgrB* gene. ND, not determined.

### Time-kill kinetics and resistance studies.

**FIG 2:**
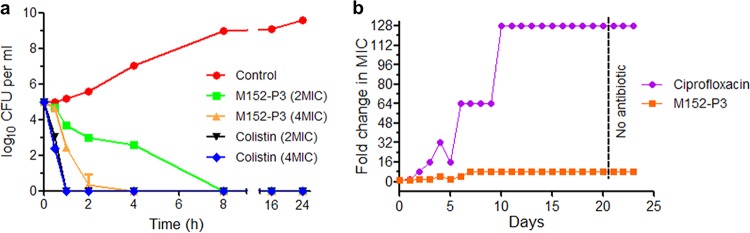
Time-kill assay of M152 and resistance acquisition study. (a) K. pneumoniae ATCC 700603 was grown in the presence of M152-P3 or colistin, and values of CFU per milliliter were determined by the plating method. M152-P3 is a slow killer, compared to colistin, but is definitely bactericidal in nature. No regrowth was observed after 24 h even with a low concentration of the peptide. The experiment was conducted in triplicate, and data are plotted as mean ± standard deviation (SD). Two biological repeats were performed, and similar results were obtained. (b) Serial passaging of K. pneumoniae with a sublethal concentration of M152-P3 or ciprofloxacin for 20 days; 3 days of subculturing without any antibiotic was then performed, to confirm the stability of resistance. M152-P3 showed a MIC increase of just 4- to 8-fold, whereas ciprofloxacin caused a 128-fold increase.

We also studied the time-dependent killing of K. pneumoniae in the presence of M152-P3 and found that the compound killed the bacteria completely within 4 to 8 h even at a very low concentration (Fig. 2a). There was no regrowth observed in time-kill assays after 24 h, which suggested the ability of this compound to resist spontaneous mutation. These results prompted us to calculate the mutation prevention concentration (MPC) and to analyze the resistance mutation frequency. M152-P3 prevented spontaneous mutation in K. pneumoniae at a concentration of 16 μg ml^−1^. The survivors that appeared under four conditions below the MPC were selected for assessment of the susceptibility to M152-P3, and none of the survivors showed resistance. Therefore, the frequency of resistance mutation was calculated as <2.5 × 10^−9^. Since the mutation frequency was very low for M152-P3, we tried to develop acquired resistance through serial passaging of bacteria for 20 days in the presence of sublethal concentrations of M152-P3 (Fig. 2b). After 20 days, the mutant showed just 4- to 8-fold increases in MIC, whereas bacteria successfully developed resistance to ciprofloxacin (128-fold changes in MIC).

### Tridecaptin M kills bacteria by blocking ATP synthesis through a cascade of reactions.

**FIG 3:**
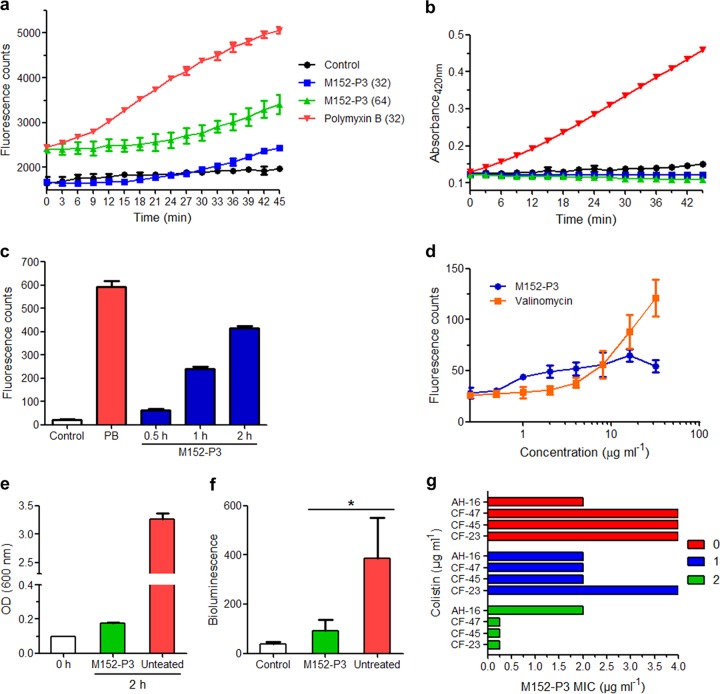
Mode-of-action studies in K. pneumoniae ATCC 700603 and synergistic activity of M152-P3 with colistin. (a) Outer membrane permeabilization assay with NPN. Values of 32 and 64 in parentheses show the concentrations, in micrograms per milliliter. An increase in fluorescence indicates the localization of NPN in the periplasm and phospholipid membrane, suggesting disruption of the outer membrane. The data are representative of three independent experiments. Data are plotted as mean ± SD of three replicates. (b) ONPG assay for large pore formation in the inner membrane. ONPG is metabolized intracellularly by β-galactosidase to produce *ortho*-nitrophenol, which absorbs at 420 nm. Polymyxin B shows inner membrane lysis, which is characteristic of this class of antibiotics. No lysis is observed in M152-P3 treated cells. Data shown here are the mean ± SD of three replicates. The experiment is representative of four independent experiments. (c) PI uptake assay. Both compounds were used at a concentration of 32 μg ml^−1^. M152-P3 shows negligible membrane lysis in 30 min, whereas polymyxin B (PB) completely lysed the cells. The data are plotted as mean ± SD of three replicates. (d) Membrane depolarization using fluorescence leakage of DiSC_3_(5). M152-P3 shows fluorescence leakage to some extent at higher concentrations. The experiment was performed with at least two biological repeats, and data are plotted as mean ± SD of three replicates of one representative. (e and f) ATP determination assays. The growth after 2 h of an untreated culture or a culture treated with a subinhibitory concentration of M152-P3 was determined (e); after treatment, the OD_600_ of the two samples was adjusted to achieve equal numbers of bacteria, and then intracellular ATP synthesis was compared by measuring bioluminescence based on the luciferase assay (f). The experiment was performed in duplicate, and the average values of three independent experiments are plotted here with SDs. Significance was calculated using a paired Student's *t* test with two-tailed distribution (*P* < 0.05). (g) Potentiation assay of M152-P3 in the presence of colistin (at the clinical breakpoint concentration). CF-23, CF-45, and CF-47 are *mcr-1*-positive E. coli strains; AH-16 is a clinical K. pneumoniae isolate. The numbers in the labels, i.e., 0, 1, and 2, are colistin concentrations, in micrograms per milliliter. In E. coli strains, the MIC of M152-P3 was reduced 16-fold with 2 μg ml^−1^ colistin, whereas no synergy was observed in AH-16. The FICIs for colistin in strain CF-23, CF-45, and CF-47 were 0.31, 0.56, and 0.56, respectively, when it was used at 2 μg ml^−1^.

The study further aimed at discerning the mode of action of M152-P3 in K. pneumoniae. Recently, Cochrane et al. showed that tridecaptin A_1_ binds specifically to lipid II of Gram-negative bacteria (25). They also reported that LPS interaction is necessary to facilitate the crossing and entry of tridecaptin into the periplasmic space (where lipid II is situated). We performed a series of *in vitro* experiments to demonstrate the mode of action of M152-P3, and we compared the results with those obtained previously for tridecaptins. In an attempt to study the effect of this compound on the outer membrane of Gram-negative bacteria, *N*-phenyl-1-naphthylamine (NPN) (35, 36) dye was used. Polymyxin B caused a rapid increase in fluorescence, compared to control cells (Fig. 3a), which indicates disruption of the outer membrane. This is a characteristic phenomenon of polymyxins in Gram-negative bacteria (27, 37, 38). In contrast, M152-P3 showed poor or negligible permeabilization at 32 μg ml^−1^, while this effect appeared to be greater at higher concentrations, which explains the higher MICs of M152-P3 versus polymyxins in polymyxin-sensitive strains. We then checked whether M152-P3 acts similarly on the inner membrane. Two membrane-impermeable substrates, i.e., *ortho*-nitrophenyl-β-galactoside (ONPG) and propidium iodide (PI), were used to observe large pore formation in the inner membrane. As visible in Fig. 3b, M152-P3 did not facilitate the entry of ONPG into the cells, whereas polymyxin B-treated cells had much greater uptake of ONPG. These data indicate that M152-P3 did not create large pores in the membrane. Similar results were obtained with PI (Fig. 3c). The rise in intensity with prolonged incubation may be due to the cell population that is already dead (25). We also studied the changes in membrane potential using localization of a voltage-sensitive fluorescent probe, i.e., 3,3-dipropylthiacarbocyanine [DiSC_3_(5)], as a proxy. In Fig. 3d, it is clearly visible that the addition of valinomycin caused fluorescence leakage in a concentration-dependent manner. This validated our assay to study the membrane potential in the presence of M152-P3. After the addition of M152-P3, fluorescence intensity was enhanced to some extent, in a concentration-dependent manner. This result was in contrast to findings reported previously for tridecaptin A_1_ (25). Cochrane et al. showed that tridecaptin A_1_ altered the proton motive force by creating proton-specific pores, and they concluded that disruption of the proton motive force would block energy generation in bacteria, ultimately causing cell death (25). We monitored the ATP synthesis in peptide-treated bacteria via a luciferase-based ATP determination assay and compared the results with those obtained for nontreated cells. After 2 h of treatment, the optical density at 600 nm (OD_600_) of bacteria increased almost 2-fold, indicating that the peptide concentration used in the assay was not lethal to cells (Fig. 3e). In contrast, the untreated sample reached an OD_600_ of approximately 3.5. This finding suggests that M152-P3 halted bacterial growth even at a subinhibitory concentration and caused a prolonged lag phase or adaptation phase. This explanation was supported by the luciferase assay results. The bacteria accumulated low levels of ATP in the presence of a subinhibitory concentration of M152-P3 (Fig. 3f), showing the slowdown of this important pathway and initial growth retardation. Other antimicrobial peptides, e.g., nigericin and subtilisin, have also been shown to create proton-specific pores in the membrane (39, 40). Therefore, inhibition of ATP synthesis is a consequence of this unregulated proton motive force caused by the action of such peptides.

As mentioned in previous reports, tridecaptins behaved similarly with LPS, compared to polymyxins (25). This was assumed to be the fundamental attack that helps them disrupt the outer membrane and reach their main target. If this is the case, however, then tridecaptins should not interact with LPS in the first place and be able to reach the target in polymyxin-resistant strains in which LPS modification is the main cause of resistance. Since tridecaptins retain superior activity despite modifications in LPS (leading to reduced binding with polymyxins), the results imply that the interactions of tridecaptins with LPS at the molecular level are different from those of polymyxins. However, further detailed investigations are required to confirm their molecular interactions with LPS and to compare the interaction site with that of polymyxins.

### Potentiation of tridecaptin M in the presence of colistin.

Considering the low outer membrane permeabilization potential of M152-P3, we postulated that this compound could exert its effect at a lower concentration if the outer membrane was compromised by other means. Teixobactin (a lipid II inhibitor in Gram-positive bacteria) also showed activity in an E. coli strain that was genetically compromised regarding the outer membrane, suggesting that the outer membrane was a probable hindrance (2). Also, many other antibiotics that are used for Gram-positive bacteria have been reported to act in Gram-negative bacteria in synergy with colistin (21). This hypothesis prompted us to check the potentiation of M152-P3 in the presence of colistin. Figure 3g explains the synergy between colistin and M152-P3 in colistin-resistant strains. Subinhibitory concentrations of colistin (also below the clinical breakpoint for resistance) reduced the MIC of M152-P3 up to 16-fold. In one strain, CF-23, the fractional inhibitory concentration index (FICI) for colistin was 0.31, which clearly demonstrates the synergy between these two classes. These results indicate that tridecaptins can be further optimized for the ability to penetrate the outer layer more efficiently.

### Therapeutic efficacy in a mouse model and toxicity studies.

**FIG 4:**
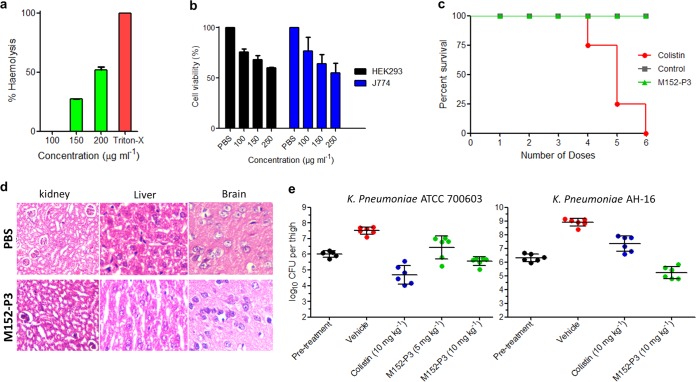
Low *in vitro* and *in vivo* toxicity of M152-P3 and efficacy in thigh infection models of colistin-resistant K. pneumoniae. (a) Hemolysis assay with rabbit RBCs. No lysis of RBCs at up to 100 μg ml^−1^ was observed. The data are plotted as mean ± SD of three replicates. (b) *In vitro* mammalian toxicity in HEK 293 and J774 cell lines. The data shown here are mean ± SD of three replicates. (c) Acute toxicity study in mice (*n* = 4). Six doses (12 mg/kg) of M152-P3 or colistin were given subcutaneously at 2-h intervals. No mouse in the colistin group could tolerate the six doses, whereas the M152-P3 group showed no deaths at the administered dose and the mice behaved similarly to the placebo group. (d) Histopathology of major organs of mice after the six-dose administration. M152-P3 showed no significant changes in any of the organs, and results were comparable to those observed with PBS. (e) *In vivo* efficacy of M152-P3 in the thigh infection model (six mice in each group). K. pneumoniae ATCC 700603 was injected intramuscularly in the right thigh, and single-dose treatment was given subcutaneously after 4 h. Values of CFU per thigh were determined 24 h postinfection. The vehicle group received PBS. In the case of colistin-resistant K. pneumoniae AH-16, treatment was given 2 h and 11 h postinfection, and the mice were sacrificed 24 h postinfection. CFU for each mouse were calculated in duplicate, and the average values are plotted. Each point in the graph represents one mouse. The data are plotted as mean ± SD.

The *in vitro* efficacy of M152-P3 in colistin- or polymyxin-resistant XDR strains prompted us to study its therapeutic potential in more detail. The hemolysis assay indicated that this compound had no effect on erythrocytes at concentrations of up to 100 μg ml^−1^, which is >25-fold higher than its MIC in bacteria (Fig. 4a). In cytotoxicity experiments as well, the compound exhibited 50% inhibitory concentration (IC_50_) values of >250 μg ml^−1^ in the human embryonic kidney (HEK) cell line HEK 293 and the mouse macrophage cell line J774 (Fig. 4b). These results are quite desirable for antimicrobial compounds to be administered systemically in humans. Although the IC_50_ values in cell lines were significantly greater than the antibacterial concentrations, it should be noted that approximately 25% cell toxicity was observed at 100 μg ml^−1^. To provide further insights into the toxicity of this compound, we examined the acute toxicity of M152-P3 in an animal model. M152-P3 or colistin was given to mice at 12 mg kg^−1^ every 2 h until an accumulated dose of 72 mg kg^−1^ had been administered. No animal in the colistin group could survive the six doses (Fig. 4c), and all of the mice died within 24 h. The results were reasonably similar to the previously observed effects of colistin, in which intravenous injection of colistin at 8.5 mg kg^−1^ showed lethality to mice (41). Also, nephrotoxicity has been associated with polymyxins given subcutaneously at the same dose as used in the present study (41, 42). In contrast, M152-P3 was well tolerated in mice, and no deaths were observed for 24 h after the last dose, at which time mice were sacrificed for histopathology experiments. The mice behaved like the placebo group (treated with phosphate-buffered saline [PBS]), and no significant changes were noted in histopathology analyses of major organs (Fig. 4d). We could not observe any nephrotoxicity for M152-P3 at the given dose. The toxicity results were more favorable, compared with colistin, in the *in vivo* system; in our previous study, colistin showed an IC_50_ in the HEK 293 cell line of >500 μg ml^−1^ (27), whereas it displayed severe toxicity in animals. This observation can be supported by the assumption that tridecaptin M may bind (reversibly or irreversibly) to serum or plasma proteins to a greater extent than colistin, and thus its pharmacokinetic (PK)/pharmacodynamic (PD) profile may be different from that of colistin. This needs to be confirmed in further studies. Finally, we wanted to know whether this compound would exert its effect *in vivo* to eradicate bacteria from the body. A thigh infection model was set up to assess the *in vivo* efficacy of M152-P3 against polymyxin-sensitive K. pneumoniae ATCC 700603, as described previously (21, 22). At 10 mg kg^−1^, colistin reduced the bacterial burden by almost 1.5 log CFU per thigh, whereas M152-P3 reduced it by ∼0.5 log CFU (Fig. 4e). These results were expected, since the *in vitro* MIC of colistin is nearly 4-fold less than that of M152-P3 against this strain. Nonetheless, this is noteworthy that M152-P3 successfully reached the tissue and showed efficacy against bacteria, compared to the untreated control. Next, we infected mice with a colistin-resistant XDR strain (AH-16) and treated them with two doses (10 mg kg^−1^) of colistin or M152-P3. Colistin failed to eradicate the bacteria in this model, and bacteria were able to multiply in the muscle, whereas M152-P3 displayed 90% removal of the bacterial population (Fig. 4e). These results indicate the excellent therapeutic efficacy of this class of antibiotics, to develop a suitable candidate against colistin-resistant *Enterobacteriaceae*.

## DISCUSSION

The dwindling antibiotic pipeline and the growing nonresponsiveness of antibiotic-resistant Gram-negative pathogens to colistin have propelled us to the verge of a “postantibiotic era.” This scenario requires our immediate actions to address these organisms by developing new drugs and to find appropriate solutions to control the spread of resistance. Natural products built our antibiotic arsenal in the golden era and continued to provide new drugs with the advancement of medicinal chemistry (1). Conventional approaches for isolating bacterial natural products soon faced the replication of already discovered compounds. No new class emerged from microbes after the 1980s until very recently, when uncultured bacteria or microbes dwelling in unusual habitats proved to be sources of novel antimicrobial entities (2, 3). The discovery of such antibiotics stimulated a resurgence in the exploitation of new natural products to fight difficult-to-address pathogens (43). The present study discusses the discovery of a new variant of the tridecaptin class that is effective against colistin-resistant Gram-negative superbugs. The producer strain, M-152, was initially discarded because of the thought that it would secrete polymyxin, because we analyzed the whole-genome sequence of a previously reported strain of P. jamilae (44) available at NCBI and observed the polymyxin biosynthetic gene cluster. This genus is very well known to produce polymyxins, which are mainly active against Gram-negative bacteria (11, 27, 45). Later, the crude extract of this strain was found to inhibit colistin-resistant and colistin-sensitive K. pneumoniae strains at similar concentrations. This curiosity turned this study into a rational discovery of tridecaptin M, belonging to a class that had not been assayed against colistin-resistant bacteria in the past. The compound showed strong antimicrobial activity against polymyxin-resistant XDR clinical isolates of K. pneumoniae and *mcr-1*-positive E. coli strains. The studies on the mode of action were consistent with previous reports (25) and thus corroborate these assays. We observed some discrepancies in membrane potential assay results. Previously, it was shown that tridecaptin A_1_ did not cause depolarization in E. coli or A. baumannii when cells treated with the anionic dye bis-(1,3-dibarbituric acid)-trimethine oxanol (DiBAC_4_) were incubated with the peptide (25, 46, 47). This dye enters depolarized cells and binds to proteins and membrane components. In contrast to DiBAC_4_, DiSC_3_(5) is a cationic dye that accumulates on polarized cells and embeds itself in the lipid membrane, where its fluorescence is masked (47, 48). If depolarization of the membrane occurs, then the accumulated dye moves back to the surrounding environment, causing an increase in fluorescence intensity. This leakage in fluorescence is a measure of the change in membrane potential. Although the change in membrane potential in M152-P3-treated cells was not as great as in case of valinomycin, this can be countered with the fact that both K^+^ and H^+^ ions are responsible for the membrane potential (47, 49, 50) and valinomycin is specific to the transport of K^+^ ions down the electrochemical gradient (51). In normal respiring cells, H^+^ ions contribute most to the membrane potential gradient, due to the active electron transport chain (49, 50). Addition of valinomycin causes movement of K^+^ ions inside the cell, thus leading to a reduction in membrane potential (also called depolarization). We thought of another possibility, i.e., an inward movement of H^+^ ions due to the formation of proton-specific pores would also lead to disturbance of the membrane potential (39, 40). This could be a plausible explanation for the change in membrane potential after M152-P3 addition to the cells. Indeed, this phenomenon was observed for tridecaptin A_1_. One explanation for negative results with DiBAC_4_ could be the interference of this dye with tridecaptins (47). Since this compound targets lipid II (which is not a protein) and ultimately makes the bacteria devoid of ATP generation, there are very few chances of developing resistance to this antibiotic. This was clearly observed in an *in vitro* experiment (Fig. 2b). Vancomycin and the recently discovered antibiotic teixobactin also target lipid II in Gram-positive bacteria and have been complemented with the ability to thwart the development of resistant mutants (2).

Tridecaptins were discovered at a time when the existence of colistin resistance was rare and other safe antibiotics were available. Also, they shared greater homology in structure with the polymyxins, which led people to think that they might possess similar nephrotoxicity; moreover, they showed decreased antibacterial activity, compared with polymyxins. These are some of the reasons this class remained underexplored over the years. Cochrane et al. (25) also found that tridecaptin A_1_ bound to LPS like polymyxins, and they might have assumed that LPS modification would restrict this class from its binding to LPS. On the contrary, the foundation of the present study was the activity of this class observed in colistin-resistant strains.

To conclude, M152-P3 appeared safe to red blood cells (RBCs) and mammalian cell lines at concentrations much higher than its effective concentration in bacteria. Also, no acute toxicity was observed in the animal model at the administered dose. Above all, the compound exhibited remarkable efficacy in a thigh infection model with a colistin-resistant strain of K. pneumoniae. Nevertheless, this compound requires detailed investigations regarding its PK and PD. Although some structure-activity relationship studies have been carried out with this class (26, 31, 52, 53), more efforts are needed to design a suitable clinical drug candidate. It is also worthwhile to study how this class can be optimized to improve the activity in other Gram-negative bacteria. Overall, we demonstrate the desirable therapeutic indices of this class of antibiotics against the toughest superbugs, which demand new and effective drug development. The study also emphasizes the significance of natural products as pioneering drugs to fight pathogens expressing resistance to even last-line antibiotics.

## MATERIALS AND METHODS

### Bacterial strain and isolation of antimicrobial compound M152-P3.

Bacterial isolates obtained from the mud sample (63) were screened for antimicrobial activity. One mud bacterium, named M-152, inhibited the growth of both Gram-negative and Gram-positive bacteria. The inoculum was prepared in 100 ml Mueller-Hinton broth (MHB), and log-phase cultures were transferred in 2-liter flasks containing 700 ml sterile MHB (2% inoculum). Fermentation was carried out in 5.6-liter batches and lasted for 24 h. The cultures were harvested by centrifugation. We prepared crude extract using Diaion HP-20 resin (27). The antimicrobial compound was partially purified by bioactivity-guided fractionation by cation-exchange chromatography, with the following conditions: SP-Sepharose column (bed height, 5 cm by 15 cm; column volume, 300 ml); flow rate, 10 ml/min; buffer, 10 mM ammonium acetate (pH 5.0 ± 0.1); 1 M NaCl in the same buffer was used as an eluent solution. A two-stage linear gradient of 0 to 0.5 M NaCl in 900 ml and 0.5 to 1 M in 300 ml was used to elute the bound components. Fractions of 100 ml were collected and assayed for antimicrobial activity against K. pneumoniae. The active fractions were further purified by reverse-phase HPLC on a C_18_ column (Phenomenex, 21.2 mm by 250 mm, 5 μm), using 20% acetonitrile in water and 100% acetonitrile (both containing 0.075% trifluoroacetic acid [TFA]) as solvent A and solvent B, respectively. The gradient was ramped from 0% solvent B to 18% solvent B in 25 min and from 18% solvent B to 80% solvent B in 11 min. All peaks were collected and assayed for bioactivity. The active peak was further purified by a second round of HPLC on a C_4_ column (Phenomenex Jupiter, 10 mm by 250 mm, 5 μm, 300 Å), with 20% acetonitrile/80% 5 mM ammonium acetate buffer (pH ∼5.2) as solvent A and 100% acetonitrile as solvent B. The gradient was ramped from 5% solvent B to 30% solvent B in 25 min. The pure compound, named M152-P3, was lyophilized after desalting, to obtain the off-white powder.

### Structural characterization by MS, amino acid analysis, and NMR spectroscopy.

M152-P3 was subjected to LC-ESI-MS, ESI-HRMS, and LC-ESI-MS/MS analysis to determine the monoisotopic mass and fragmentation pattern. The daughter ion series generated in the MS/MS analyses were assigned manually, and *de novo* sequencing was performed to deduce the probable amino acid sequence. For amino acid analysis, 100 μg of the compound was hydrolyzed and derivatized according to the manufacturer’s protocol (PicoTag amino acid analysis system; Waters). The hydrolysate was analyzed by HPLC, and the retention times of amino acids were compared with those of standard amino acids according to the protocol. For NMR spectroscopy, 10 to 12 mg of pure compound was dissolved in deuterated dimethyl sulfoxide, and ^1^H, ^13^C, COSY, TOCSY, distortionless enhancement by polarization transfer [DEPT], HMBC, and HSQC spectra were acquired on a Bruker 600-MHz spectrometer.

### Marfey’s analysis.

Marfey’s analysis with Marfey’s reagent (l-1-fluoro-2-4-dinitrophenyl-5-l-alanine amide [FDAA]) (product no. 48895; Thermo Scientific) was performed to determine the stereochemistry of the amino acids. Three milligrams of the compound was hydrolyzed to its amino acid constituents and derivatized according to the manufacturer’s instructions. For detection of FDAA derivatives of amino acids, reverse-phase HPLC (Waters XBridge C_18_, 4.6 by 250 mm, 5 μm, 130 Å) was employed, with the following mobile phase system: solvent A, water containing 0.075% TFA; solvent B, acetonitrile containing 0.075% TFA. A linear gradient from 18% solvent B to 46% solvent B in 60 min was used. The retention times of standard amino acids were compared with those obtained in the M152-P3 chromatogram.

### Biosynthetic gene cluster identification.

The whole-genome sequence was obtained by using a hybrid assembly of Illumina reads and Nanopore data. The biosynthetic gene cluster was located with the help of antiSMASH 3.0 (54). The preference of the adenylation domain for specific amino acids was checked using NRPSpredictor2 and other programs provided in antiSMASH, and data were curated manually with the help of experimentally determined structures.

### MIC determination.

MICs were determined using the broth microdilution assay in CA-MHB, according to CLSI guidelines (64). Briefly, the bacterial cell concentration was adjusted to approximately 4 × 10^5^ CFU/ml. The experiment was performed in a 96-well polystyrene tissue culture plate (product no. 353072; Falcon), in 200 μl. The bacteria were incubated for 18 h at 37°C with 2-fold varying concentrations of M152-P3. The lowest concentration with no visible growth was considered the MIC. The standard ATCC cultures were procured from HiMedia Laboratory (India). The antibiotic susceptibility of all clinical isolates was examined using either the disk diffusion method or the broth microdilution method, according to CLSI guidelines. The data are given in the supplemental material, with references to previous studies from our group for some isolates.

### Time-dependent killing.

K. pneumoniae ATCC 700603 was grown to log phase, and the OD_600_ was adjusted to 0.35 (4 × 10^8^ CFU ml^−1^). This culture was diluted 1,000-fold in fresh MHB with 2×MIC or 4×MIC concentrations of M152-P3 or colistin and was incubated at 37°C. Fifty to 100 μl of the culture was spread plated on Mueller-Hinton agar (MHA) plates at different time points. The culture without antimicrobial compound was taken as the positive control. CFU per milliliter values were calculated and plotted against time. The experiment was performed in triplicate, with two biological repeats.

### MPC and resistance frequency analysis.

K. pneumoniae ATCC 700603 was grown to log phase and adjusted to an OD_600_ of ∼1.0 (10^9^ CFU ml^−1^). One hundred microliters of this culture was plated on cation-adjusted MHA plates supplemented with different concentrations of M152-P3 (0.5×MIC, 1.0×MIC, 1.5×MIC, 2.0×MIC, and 4×MIC). The plates were incubated at 37°C for 24 h. The plates were observed for survivors, and the concentration at which no colony appeared was considered the MPC. To calculate the resistance mutation frequency, the survivors that appeared after 24 h (at lower concentrations) were replated (two colonies from each concentration) on a fresh agar plate with a concentration of M152-P3 similar to that from which the survivor emerged. After reconfirmation of the survivors, 4 to 6 representatives from each condition were assayed for their susceptibility to M152-P3. A survivor was considered resistant if the MIC increased a minimum of 4-fold, since a 2-fold change in MIC can occur due to intrinsic variations in MIC determinations (2). The mutation frequency was determined (55) according to the following equation: frequency of resistance = number of resistant mutants/(number of conditions × CFU plated).

### Resistance studies.

To study the development of acquired resistance to M152-P3, we conducted sequential passaging (2, 25) of K. pneumoniae ATCC 700603 in the presence of sublethal concentrations of M152-P3 or ciprofloxacin. Four different concentrations, i.e., 0.25×MIC, 0.5×MIC, 1×MIC, and 2×MIC, in CA-MHB were used to grow the cultures for 24 h. The following day, the highest concentration showing a minimum OD_600_ of ≥0.3 was transferred (3 to 4 μl in 200 μl of broth in a 96-well plate) into fresh broth containing higher concentrations of antibacterial compounds. This sequential culturing was performed for 20 days with antibiotic and then 3 days without antibiotic, to check the stability of acquired resistance. MICs were determined every day for M152-P3 or ciprofloxacin, and fold changes in MICs were measured.

### Outer membrane permeabilization assay.

For outer membrane permeabilization, the NPN assay was used (27). K. pneumoniae ATCC 700603 cells were grown in MHB and washed twice with 5 mM HEPES buffer (containing 5 mM glucose [pH ∼7.4]). The final cell concentration was adjusted to an OD_600_ of 0.06. NPN was dissolved in acetone at a concentration of 20 mM and then diluted in HEPES buffer to make a working stock of 40 μM. One hundred thirty microliters of cells were incubated with 50 μl of NPN (final concentration, 10 μM) and 20 μl of M152-P3 or polymyxin B at different concentrations (0.5 to 64 μg ml^−1^). The wells without antibiotic were taken as the negative control. Polymyxin B was used as a standard because of its strong membrane-permeabilizing properties. The fluorescence was measured in a time-dependent manner on a fluorescence microplate reader (Synergy H1; Biotek), with excitation at 350 nm and emission at 420 nm. NPN plus antibiotic without cells was also assayed, to check the fluorescence interference. The experiment was performed in triplicate, with two biological repeats.

### Inner membrane permeabilization assay.

For inner membrane permeabilization, the ONPG assay was performed (56). K. pneumoniae was grown in lactose medium, and the cells were washed twice with PBS and adjusted to an OD_600_ of 0.05 (5 × 10^7^ CFU/ml). One hundred eighty microliters of cells was incubated with 1.5 mM ONPG and 20 μl of M152-P3 or polymyxin B, at different concentrations, in a 96-well plate at 37°C. The absorbance was measured at 420 nm on a microplate reader (BioTek) from 0 to 45 min, at 3-min intervals. The experiment was conducted in triplicate. Cells without peptide were considered the negative control. Three biological repeats were performed. Cytoplasmic membrane permeabilization was also studied with a membrane-impermeable fluorescence dye, PI (Thermo Fisher Scientific). The protocol mentioned in the LIVE/DEAD BacLight bacterial viability kit (product no. L7012; Molecular Probes [purchased from Thermo Fisher Scientific]) was optimized initially with some modifications. Bacterial cells were grown in MHB and washed three times with normal saline (0.9% NaCl). The cell concentration was normalized to 10^8^ CFU/ml or an OD_600_ of 0.1. The cells were treated with different concentrations of M152-P3 or polymyxin B in a 1.5-ml microcentrifuge tube. After 30 min, PI was added at a final concentration of 2.5 μg/ml, and cells were kept in the dark at 4°C for 15 to 20 min. The samples were centrifuged at 10, 000 × *g* for 10 min to remove the unbound dye and excess antibiotics. The cells were resuspended in 350 μl of normal saline and mixed well. The cells (100 μl per well) were dispensed in a 96-well plate (suitable for fluorescence measurements), and the fluorescence was measured on a fluorescence microplate reader, with excitation at 535 nm and emission at 617 nm. The experiment was conducted in triplicate, and at least three biological repeats were performed. The cells without any treatment were considered as the PI-negative control.

### DiSC_3_(5) assay.

Membrane depolarization was studied using the membrane potential-sensitive probe DiSC_3_(5) (Sigma-Aldrich) (57–59). K. pneumoniae cells were prepared as described for the NPN assay. The cells were incubated with DiSC_3_ dye (0.4 μM) and 100 mM KCl in a 96-well black plate (total volume, 180 μl) and checked for stable reduction in fluorescence, as measured with a microplate reader. Cells were incubated with KCl to equilibrate the cytoplasmic and external K^+^ concentrations; this was followed by the addition of different concentrations of M152-P3 or polymyxin B (20 μl) and incubation for 30 min. The fluorescence leakage was measured with excitation at 622 nm and emission at 670 nm. The experiment was performed in triplicate.

### ATP determination.

K. pneumoniae ATCC 700603 was grown to mid-log phase and inoculated in 5 ml LB broth in 100-ml Erlenmeyer flasks to achieve 5 × 10^7^ CFU/ml. The medium contained 10 mM glucose and M152-P3 at 16 μg ml^−1^. The flasks were incubated at 37°C at 200 rpm. Control flasks contained no antibiotic. Samples (2 ml) from each flask were taken at 2 h, and the OD_600_ was adjusted to obtain similar values of CFU per milliliter for each set (variations in the number of bacteria may interfere with the results). The culture was pelleted and washed with PBS. The pellet was dissolved in lysis buffer (500 μl), and the cells were lysed using either an ultrasonic water bath for 15 min at 4°C or the freeze-thaw method. In the freeze-thaw method, the pellet was frozen at −80°C for 15 min and immediately kept in boiling water for 15 min. The cell lysate was centrifuged at 4°C, and the supernatant was assayed for total intracellular ATP synthesis in treated and nontreated bacteria using an ATP determination kit (product no. A22066; Invitrogen, Molecular Probes).

### M152-P3 potentiation assay.

E. coli strains CF-23, CF-45, and CF-47 (*mcr-1* positive) and K. pneumoniae strain AH-16 were used to study synergy between M152-P3 and colistin. Colistin was used at concentrations below its clinical breakpoint (≤2 μg ml^−1^), and M152-P3 was diluted in a 2-fold manner. The fold modulation was calculated as the reduction in M152-P3 MIC with versus without colistin.

### Hemolysis and mammalian cytotoxicity.

Hemolysis experiments were performed as described previously (60) Briefly, fresh rabbit RBCs were washed three times with PBS and resuspended in PBS at a concentration of 4% (vol/vol). RBCs (180 μl) were added to the wells of a 96-well U-bottom plate; 20 μl of M152-P3 was added, resulting in final concentrations ranging from 0 to 200 μg ml^−1^. After 1 h of incubation at 37°C, cells were centrifuged at 1,000 × *g*, and 100 μl of the supernatant was transferred to a 96-well flat-bottom plate. The release of hemoglobin was measured as the absorbance at 570 nm, in the microplate reader. The experiment was performed in triplicate, with two biological repeats. Mammalian cytotoxicity was determined with two different cell lines, i.e., the HEK cell line HEK 293 and the mouse macrophage cell line J774 (63). The cells were treated with M152-P3 for 24 h at concentrations ranging from 0 to 250 μg ml^−1^.

### Animal studies.

All animal experiments were conducted at the animal facility of the CSIR-Institute of Microbial Technology and were in compliance with the ethical standards of the Institutional Animal Ethics Committee (IAEC) of the CSIR-Institute of Microbial Technology (approval IAEC/17/11). Female BALB/c mice (6 to 8 weeks of age) were used in all studies. Randomization or blinding was not deemed necessary in efficacy experiments. In histopathology analyses, the observer was unaware of the treatment groups.

### *In vivo* toxicity.

*In vivo* toxicity was studied as described previously for polymyxins (42). Mice were injected subcutaneously with M152-P3 or colistin, at a dose of 12 mg/kg, in normal saline. Six doses were given, at 2-h intervals, until a final accumulation of 72 mg/kg had been administered. Twenty hours after the last dose, mice were sacrificed and all major organs (i.e., kidneys, spleen, liver, and brain) were isolated and fixed in 10% buffered formalin (pH 7.2). The organs were submitted for histological staining. Four mice were taken for each group. One group received only normal saline (vehicle control). All of the mice were observed for survival until euthanization, since colistin-receiving mice died before the last dose. The data were presented as any changes in the kidneys or other organs and as Kaplan-Meier survival plots (61), in terms of acute toxicity.

### Mouse thigh infection model.

The *in vivo* efficacy of M152-P3 against K. pneumoniae strain ATCC 700603 (polymyxin sensitive) and K. pneumoniae clinical isolate AH-16 (polymyxin resistant and XDR) was assessed in a mouse thigh infection model (22). Female BALB/c mice (*n* = 6) were rendered neutropenic with two doses of cyclophosphamide, i.e., 150 mg/kg and 100 mg/kg, administered 4 days and 1 day prior to infection, respectively. The mice were then injected intramuscularly, in the right thigh muscle, with 5 × 10^5^ CFU per mouse. At 4 h postinfection, all of the mice were treated subcutaneously with colistin or M152-P3 (5 or 10 mg/kg). The control group received vehicle (normal saline). At 20 h after treatment, mice were sacrificed and CFU in muscle were determined on MHA plates through serial dilution in PBS after homogenization of muscle tissue. In the infection model with the AH-16 strain, two doses (10 mg/kg) were given for both colistin and M152-P3, at 2 and 11 h postinfection. At 20 h after the first dose, the mice were sacrificed and CFU per thigh were calculated as described above.

### Data availability

The whole-genome sequence of the producer strain M152 has been deposited in GenBank under accession number CP034141. Additional data that support the findings of this study are available from the corresponding author upon request.

## Supplementary Material

Supplemental file 1

## References

[1] Brown ED, Wright GD 2016 Antibacterial drug discovery in the resistance era. Nature 529:336. doi:10.1038/nature17042. 26791724

[2] Ling LL, Schneider T, Peoples AJ, Spoering AL, Engels I, Conlon BP, Mueller A, Schäberle TF, Hughes DE, Epstein S, Jones M, Lazarides L, Steadman VA, Cohen DR, Felix CR, Fetterman KA, Millett WP, Nitti AG, Zullo AM, Chen C, Lewis K 2015 A new antibiotic kills pathogens without detectable resistance. Nature 517:455. doi:10.1038/nature14098. 25561178 PMC7414797

[3] Zipperer A, Konnerth MC, Laux C, Berscheid A, Janek D, Weidenmaier C, Burian M, Schilling NA, Slavetinsky C, Marschal M, Willmann M, Kalbacher H, Schittek B, Brötz-Oesterhelt H, Grond S, Peschel A, Krismer B 2016 Human commensals producing a novel antibiotic impair pathogen colonization. Nature 535:511. doi:10.1038/nature18634. 27466123

[4] Chellat MF, Riedl R 2017 Pseudouridimycin: the first nucleoside analogue that selectively inhibits bacterial RNA polymerase. Angew Chem Int Ed Engl 56:13184–13186. doi:10.1002/anie.201708133. 28895263

[5] Hover BM, Kim S-H, Katz M, Charlop-Powers Z, Owen JG, Ternei MA, Maniko J, Estrela AB, Molina H, Park S, Perlin DS, Brady SF 2018 Culture-independent discovery of the malacidins as calcium-dependent antibiotics with activity against multidrug-resistant Gram-positive pathogens. Nat Microbiol 3:415. doi:10.1038/s41564-018-0110-1. 29434326 PMC5874163

[6] Pew Charitable Trusts. 2018 Antibiotics currently in global clinical development. Pew Charitable Trusts, Philadelphia, PA https://www.pewtrusts.org/-/media/assets/2018/09/antibiotics_currently_in_global_clinical_development_sept2018.pdf?la=en&hash=BDE8590154A21A3167CB62A80D663534906C4308.

[7] Tacconelli E, Carrara E, Savoldi A, Harbarth S, Mendelson M, Monnet DL, Pulcini C, Kahlmeter G, Kluytmans J, Carmeli Y, Ouellette M, Outterson K, Patel J, Cavaleri M, Cox EM, Houchens CR, Grayson ML, Hansen P, Singh N, Theuretzbacher U, Magrini N 2018 Discovery, research, and development of new antibiotics: the WHO priority list of antibiotic-resistant bacteria and tuberculosis. Lancet Infect Dis 18:318–327. doi:10.1016/S1473-3099(17)30753-3. 29276051

[8] Krapp F, Ozer EA, Qi C, Hauser AR 2018 Case report of an extensively drug-resistant *Klebsiella pneumoniae* infection with genomic characterization of the strain and review of similar cases in the United States. Open Forum Infect Dis 5:ofy074. doi:10.1093/ofid/ofy074. 29876363 PMC5961207

[9] Bathoorn E, Tsioutis C, da Silva Voorham J, Scoulica E, Ioannidou E, Zhou K, Rossen J, Gikas A, Friedrich A, Grundmann H 2016 Emergence of pan-resistance in KPC-2 carbapenemase-producing *Klebsiella pneumoniae* in Crete, Greece: a close call. J Antimicrob Chemother 71:1207–1212. doi:10.1093/jac/dkv467. 26817488

[10] Bi W, Liu H, Dunstan RA, Li B, Torres VVL, Cao J, Chen L, Wilksch JJ, Strugnell RA, Lithgow T, Zhou T 2017 Extensively drug-resistant *Klebsiella pneumoniae* causing nosocomial bloodstream infections in China: molecular investigation of antibiotic resistance determinants, informing therapy, and clinical outcomes. Front Microbiol 8:1230. doi:10.3389/fmicb.2017.01230. 28713357 PMC5492486

[11] Landman D, Georgescu C, Martin DA, Quale J 2008 Polymyxins revisited. Clin Microbiol Rev 21:449–465. doi:10.1128/CMR.00006-08. 18625681 PMC2493081

[12] Newton B 1956 The properties and mode of action of the polymyxins. Bacteriol Rev 20:14. 13303920 10.1128/br.20.1.14-27.1956 PMC180843

[13] Davies M, Walsh TR 2018 A colistin crisis in India. Lancet Infect Dis 18:256–257. doi:10.1016/S1473-3099(18)30072-0. 29396009

[14] Olaitan AO, Morand S, Rolain J-M 2014 Mechanisms of polymyxin resistance: acquired and intrinsic resistance in bacteria. Front Microbiol 5:643. doi:10.3389/fmicb.2014.00643. 25505462 PMC4244539

[15] Wright MS, Suzuki Y, Jones MB, Marshall SH, Rudin SD, Van Duin D, Kaye K, Jacobs MR, Bonomo RA, Adams MD 2015 Genomic and transcriptomic analyses of colistin-resistant clinical isolates of *Klebsiella pneumoniae* reveal multiple pathways of resistance. Antimicrob Agents Chemother 59:536–543. doi:10.1128/AAC.04037-14. 25385117 PMC4291396

[16] Jaidane N, Bonnin RA, Mansour W, Girlich D, Creton E, Cotellon G, Chaouch C, Boujaafar N, Bouallegue O, Naas T 2018 Genomic insights into colistin-resistant *Klebsiella pneumoniae* from a Tunisian teaching hospital. Antimicrob Agents Chemother 62:e01601-17. doi:10.1128/AAC.01601-17. 29229634 PMC5786813

[17] Baron S, Hadjadj L, Rolain J-M, Olaitan AO 2016 Molecular mechanisms of polymyxin resistance: knowns and unknowns. Int J Antimicrob Agents 48:583–591. doi:10.1016/j.ijantimicag.2016.06.023. 27524102

[18] Liu Y-Y, Wang Y, Walsh TR, Yi L-X, Zhang R, Spencer J, Doi Y, Tian G, Dong B, Huang X, Yu L-F, Gu D, Ren H, Chen X, Lv L, He D, Zhou H, Liang Z, Liu J-H, Shen J 2016 Emergence of plasmid-mediated colistin resistance mechanism MCR-1 in animals and human beings in China: a microbiological and molecular biological study. Lancet Infect Dis 16:161–168. doi:10.1016/S1473-3099(15)00424-7. 26603172

[19] Sonnevend Á, Ghazawi A, Hashmey R, Haidermota A, Girgis S, Alfaresi M, Omar M, Paterson DL, Zowawi HM, Pál T 2017 Multihospital occurrence of pan-resistant *Klebsiella pneumoniae* sequence type 147 with an IS*Ecp1*-directed *bla*_OXA-181_ insertion into the *mgrB* gene in the United Arab Emirates. Antimicrob Agents Chemother 61:e00418-17. doi:10.1128/AAC.00418-17. 28438945 PMC5487649

[20] Chen L, Todd R, Kiehlbauch J, Walters M, Kallen A 2017 Notes from the field: pan-resistant New Delhi metallo-beta-lactamase-producing Klebsiella pneumoniae — Washoe County, Nevada, 2016. MMWR Morb Mortal Wkly Rep 66:33. doi:10.15585/mmwr.mm6601a7. 28081065 PMC5687261

[21] MacNair CR, Stokes JM, Carfrae LA, Fiebig-Comyn AA, Coombes BK, Mulvey MR, Brown ED 2018 Overcoming *mcr-1* mediated colistin resistance with colistin in combination with other antibiotics. Nat Commun 9:458. doi:10.1038/s41467-018-02875-z. 29386620 PMC5792607

[22] Smith PA, Koehler MFT, Girgis HS, Yan D, Chen Y, Chen Y, Crawford JJ, Durk MR, Higuchi RI, Kang J, Murray J, Paraselli P, Park S, Phung W, Quinn JG, Roberts TC, Rougé L, Schwarz JB, Skippington E, Wai J, Xu M, Yu Z, Zhang H, Tan M-W, Heise CE 2018 Optimized arylomycins are a new class of Gram-negative antibiotics. Nature 561:189. doi:10.1038/s41586-018-0483-6. 30209367

[23] Shoji JI, Hinoo H, Sakazaki R, Kato T, Wakisaka Y, Mayama M, Matsuura S, Miwa H 1978 Isolation of tridecaptins A, B and C. J Antibiot (Tokyo) 31:646–651. doi:10.7164/antibiotics.31.646. 690000

[24] Kato T, Hinoo H, Shoji JI 1978 The structure of tridecaptin A. J Antibiot (Tokyo) 31:652–661. doi:10.7164/antibiotics.31.652. 690001

[25] Cochrane SA, Findlay B, Bakhtiary A, Acedo JZ, Rodriguez-Lopez EM, Mercier P, Vederas JC 2016 Antimicrobial lipopeptide tridecaptin A_1_ selectively binds to Gram-negative lipid II. Proc Natl Acad Sci U S A 113:11561–11566. doi:10.1073/pnas.1608623113. 27688760 PMC5068289

[26] Cochrane SA, Lohans CT, van Belkum MJ, Bels MA, Vederas JC 2015 Studies on tridecaptin B_1_, a lipopeptide with activity against multidrug resistant Gram-negative bacteria. Org Biomol Chem 13:6073–6081. doi:10.1039/c5ob00780a. 25959079

[27] Jangra M, Randhawa HK, Kaur M, Srivastava A, Maurya N, Patil PP, Jaswal P, Arora A, Patil PB, Raje M, Nandanwar H 2018 Purification, characterization and in vitro evaluation of polymyxin A from *Paenibacillus dendritiformis*: an underexplored member of the polymyxin family. Front Microbiol 9:2864. doi:10.3389/fmicb.2018.02864. 30532748 PMC6265310

[28] Zhang J, Xin L, Shan B, Chen W, Xie M, Yuen D, Zhang W, Zhang Z, Lajoie GA, Ma B 2012 PEAKS DB: de novo sequencing assisted database search for sensitive and accurate peptide identification. Mol Cell Proteomics 11:M111.010587. doi:10.1074/mcp.M111.010587. PMC3322562 22186715

[29] Kato T, Sakazaki R, Hinoo H, Shoji JI 1979 The structures of tridecaptins B and C. J Antibiot (Tokyo) 32:305–312. doi:10.7164/antibiotics.32.305. 468717

[30] Kaneda T 1967 Fatty acids in the genus *Bacillus*. I. Iso-and anteiso-fatty acids as characteristic constituents of lipids in 10 species. J Bacteriol 93:894–903. 4960925 10.1128/jb.93.3.894-903.1967 PMC276533

[31] Cochrane SA, Lohans CT, Brandelli JR, Mulvey G, Armstrong GD, Vederas JC 2014 Synthesis and structure-activity relationship studies of N-terminal analogues of the antimicrobial peptide tridecaptin A_1_. J Med Chem 57:1127–1131. doi:10.1021/jm401779d. 24479847

[32] Lohans CT, van Belkum MJ, Cochrane SA, Huang Z, Sit CS, McMullen LM, Vederas JC 2014 Biochemical, structural, and genetic characterization of tridecaptin A_1_, an antagonist of *Campylobacter jejuni*. Chembiochem 15:243–249. doi:10.1002/cbic.201300595. 24382692

[33] Keating TA, Marshall CG, Walsh CT, Keating AE 2002 The structure of VibH represents nonribosomal peptide synthetase condensation, cyclization and epimerization domains. Nat Struct Biol 9:522–526. doi:10.1038/nsb810. 12055621

[34] Li J, Jensen SE 2008 Nonribosomal biosynthesis of fusaricidins by *Paenibacillus polymyxa* PKB1 involves direct activation of a d-amino acid. Chem Biol 15:118–127. doi:10.1016/j.chembiol.2007.12.014. 18291316

[35] Loh B, Grant C, Hancock R 1984 Use of the fluorescent probe 1-*N*-phenylnaphthylamine to study the interactions of aminoglycoside antibiotics with the outer membrane of *Pseudomonas aeruginosa*. Antimicrob Agents Chemother 26:546–551. doi:10.1128/AAC.26.4.546. 6440475 PMC179961

[36] Muheim C, Götzke H, Eriksson AU, Lindberg S, Lauritsen I, Nørholm MH, Daley DO 2017 Increasing the permeability of *Escherichia coli* using MAC13243. Sci Rep 7:17629. doi:10.1038/s41598-017-17772-6. 29247166 PMC5732295

[37] Tsubery H, Ofek I, Cohen S, Eisenstein M, Fridkin M 2002 Modulation of the hydrophobic domain of polymyxin B nonapeptide: effect on outer-membrane permeabilization and lipopolysaccharide neutralization. Mol Pharmacol 62:1036–1042. doi:10.1124/mol.62.5.1036. 12391265

[38] Yu Z, Qin W, Lin J, Fang S, Qiu J 2015 Antibacterial mechanisms of polymyxin and bacterial resistance. BioMed Res Int 2015:679109. doi:10.1155/2015/679109. 25664322 PMC4312571

[39] Noll KS, Sinko PJ, Chikindas ML 2011 Elucidation of the molecular mechanisms of action of the natural antimicrobial peptide subtilosin against the bacterial vaginosis-associated pathogen *Gardnerella vaginalis*. Probiotics Antimicrob Proteins 3:41–47. doi:10.1007/s12602-010-9061-4. 21949544 PMC3176456

[40] Ahmed S, Booth IR 1983 The use of valinomycin, nigericin and trichlorocarbanilide in control of the protonmotive force in *Escherichia coli* cells. Biochem J 212:105–112. doi:10.1042/bj2120105. 6307285 PMC1152016

[41] Cui A-L, Hu X-X, Gao Y, Jin J, Yi H, Wang X-K, Nie T-Y, Chen Y, He Q-Y, Guo H-F, Jiang J-D, You X-F, Li Z-R 2018 Synthesis and bioactivity investigation of the individual components of cyclic lipopeptide antibiotics. J Med Chem 61:1845–1857. doi:10.1021/acs.jmedchem.7b01367. 29412662

[42] Roberts KD, Azad MA, Wang J, Horne AS, Thompson PE, Nation RL, Velkov T, Li J 2015 Antimicrobial activity and toxicity of the major lipopeptide components of polymyxin B and colistin: last-line antibiotics against multidrug-resistant Gram-negative bacteria. ACS Infect Dis 1:568–575. doi:10.1021/acsinfecdis.5b00085. 27525307 PMC4980087

[43] Clardy J, Fischbach MA, Walsh CT 2006 New antibiotics from bacterial natural products. Nat Biotechnol 24:1541. doi:10.1038/nbt1266. 17160060

[44] Midha S, Bansal K, Sharma S, Kumar N, Patil PP, Chaudhry V, Patil PB 2015 Genomic resource of rice seed associated bacteria. Front Microbiol 6:1551. doi:10.3389/fmicb.2015.01551. 26793183 PMC4707233

[45] Shaheen M, Li J, Ross AC, Vederas JC, Jensen SE 2011 *Paenibacillus polymyxa* PKB1 produces variants of polymyxin B-type antibiotics. Chem Biol 18:1640–1648. doi:10.1016/j.chembiol.2011.09.017. 22195566

[46] Vila-Farres X, Chu J, Inoyama D, Ternei MA, Lemetre C, Cohen LJ, Cho W, Reddy BVB, Zebroski HA, Freundlich JS, Perlin DS, Brady SF 2017 Antimicrobials inspired by nonribosomal peptide synthetase gene clusters. J Am Chem Soc 139:1404–1407. doi:10.1021/jacs.6b11861. 28055186 PMC7163397

[47] Te Winkel JD, Gray DA, Seistrup KH, Hamoen LW, Strahl H 2016 Analysis of antimicrobial-triggered membrane depolarization using voltage sensitive dyes. Front Cell Dev Biol 4:29. doi:10.3389/fcell.2016.00029. 27148531 PMC4829611

[48] Ehrenberg B, Montana V, Wei M, Wuskell J, Loew L 1988 Membrane potential can be determined in individual cells from the nernstian distribution of cationic dyes. Biophys J 53:785–794. doi:10.1016/S0006-3495(88)83158-8. 3390520 PMC1330255

[49] Mitchell P 1961 Coupling of phosphorylation to electron and hydrogen transfer by a chemi-osmotic type of mechanism. Nature 191:144–148. doi:10.1038/191144a0. 13771349

[50] Saraste M 1999 Oxidative phosphorylation at the fin de siecle. Science 283:1488–1493. doi:10.1126/science.283.5407.1488. 10066163

[51] Shapiro HM 1994 Cell membrane potential analysis. Methods Cell Biol 41:121–133. doi:10.1016/S0091-679X(08)61713-6. 7532258

[52] Cochrane SA, Findlay B, Vederas JC, Ratemi ES 2014 Key residues in octyl‐tridecaptin A_1_ analogues linked to stable secondary structures in the membrane. Chembiochem 15:1295–1299. doi:10.1002/cbic.201402024. 24816483

[53] Ballantine RD, Li Y-X, Qian P-Y, Cochrane SA 2018 Rational design of new cyclic analogues of the antimicrobial lipopeptide tridecaptin A_1_. Chem Commun (Camb) 54:10634–10637. doi:10.1039/C8CC05790G. 30179243 PMC6146376

[54] Weber T, Blin K, Duddela S, Krug D, Kim HU, Bruccoleri R, Lee SY, Fischbach MA, Müller R, Wohlleben W, Breitling R, Takano E, Medema MH 2015 antiSMASH 3.0: a comprehensive resource for the genome mining of biosynthetic gene clusters. Nucleic Acids Res 43:W237–W243. doi:10.1093/nar/gkv437. 25948579 PMC4489286

[55] Stokes JM, MacNair CR, Ilyas B, French S, Côté J-P, Bouwman C, Farha MA, Sieron AO, Whitfield C, Coombes BK, Brown ED 2017 Pentamidine sensitizes Gram-negative pathogens to antibiotics and overcomes acquired colistin resistance. Nat Microbiol 2:17028. doi:10.1038/nmicrobiol.2017.28. 28263303 PMC5360458

[56] Sun C, Li Y, Cao S, Wang H, Jiang C, Pang S, Hussain M, Hou J 2018 Antibacterial activity and mechanism of action of bovine lactoferricin derivatives with symmetrical amino acid sequences. Int J Mol Sci 19:2951. doi:10.3390/ijms19102951. PMC6213309 30262770

[57] Wu M, Maier E, Benz R, Hancock RE 1999 Mechanism of interaction of different classes of cationic antimicrobial peptides with planar bilayers and with the cytoplasmic membrane of *Escherichia coli*. Biochemistry 38:7235–7242. doi:10.1021/bi9826299. 10353835

[58] Morin N, Lanneluc I, Connil N, Cottenceau M, Pons AM, Sablé S 2011 Mechanism of bactericidal activity of microcin L in *Escherichia coli* and *Salmonella enterica*. Antimicrob Agents Chemother 55:997–1007. doi:10.1128/AAC.01217-10. 21189348 PMC3067116

[59] Cheng M, Huang JX, Ramu S, Butler MS, Cooper MA 2014 Ramoplanin at bactericidal concentrations induces bacterial membrane depolarization in *Staphylococcus aureus*. Antimicrob Agents Chemother 58:6819–6827. doi:10.1128/AAC.00061-14. 25182650 PMC4249368

[60] Stark M, Liu L-P, Deber CM 2002 Cationic hydrophobic peptides with antimicrobial activity. Antimicrob Agents Chemother 46:3585–3590. doi:10.1128/AAC.46.11.3585-3590.2002. 12384369 PMC128737

[61] Goel MK, Khanna P, Kishore J 2010 Understanding survival analysis: Kaplan-Meier estimate. Int J Ayurveda Res 1:274. doi:10.4103/0974-7788.76794. 21455458 PMC3059453

[62] Ghafur A, Shankar C, GnanaSoundari P, Venkatesan M, Mani D, Thirunarayanan M, Veeraraghavan B 2019 Detection of chromosomal and plasmid-mediated mechanisms of colistin resistance in *Escherichia coli* and *Klebsiella pneumoniae* from Indian food samples. J Glob Antimicrob Resist 16:48–52. doi:10.1016/j.jgar.2018.09.005. 30244040

[63] Jangra M, Kaur M, Nandanwar H 27 3 2019 In vitro studies on a natural lantibiotic, paenibacillin: a new-generation antibacterial drug candidate to overcome multidrug-resistance. Int J Antimicrob Agents doi:10.1016/j.ijantimicag.2019.03.020. 30928682

[64] CLSI. 2015 Performance standards for antimicrobial susceptibility testing; 25th informational supplement. CLSI M100-S25. Clinical and Laboratory Standards Institute, Wayne, PA.

