# Supplementary material for: Tridecaptin M, a New Variant Discovered in Mud Bacterium, Shows Activity against Colistin- and Extremely Drug-Resistant Enterobacteriaceae
Source: Antimicrob Agents Chemother. 2019 May 23;63(6):e00338-19. doi: 10.1128/AAC.00338-19 (PMC6535564; doi:10.1128/AAC.00338-19)
Supplement: Supplemental file 1 [file AAC.00338-19-s0001.pdf]

Supplementary Information

Discovery of a new variant, Tridecaptin M in mud bacterium: End game for colistin- and extremely drug-resistant Enterobacteriaceae

Manoj Jangra[1], Manpreet Kaur[1], Rushikesh Tambat[1], Rohit Rana[2,5], Sushil K. Maurya[2,5], Neeraj Khatri[3,5], Abdul Ghafur[4], Hemraj Nandanwar[1,5*]

[1]Clinical Microbiology & Bioactive Screening Laboratory, CSIR-Institute of Microbial Technology, Sector -39A, Chandigarh, 160 036, India

[2]Natural Product Chemistry & Process Development Division, CSIR-Institute of Himalayan Bioresource Technology, Palampur, Himachal Pradesh, 176 061, India

[3]Animal house facility, CSIR-Institute of Microbial Technology, Sector -39A, Chandigarh, India

[4]Infectious Diseases, Apollo Speciality Hospitals, No. 320, Padma Complex, Teynampet, Chennai-600 035, Tamilnadu, India

[5]Academy of Scientific and Innovative Research, CSIR-HRDC, Ghaziabad, UP 201 002, India

*__Address for correspondence__

Dr Hemraj Nandanwar

Clinical Microbiology & Bioactive Screening Laboratory, CSIR - Institute of Microbial Technology, Sector -39A, Chandigarh, India, 160 036

E-mail: hemraj@imtech.res.in

Telephone: +91-172-6665338 Fax: +91-172-2690585/2690632

Supplementary tables

Table-S1 NMR assignments of all protons and carbons in M152-P3

Tridecaptin M (M152-P3)*		
**Position**	**δ^{13}C**	**δ^{1}H (multi.., J in Hz,)**
1	174.0	----
2	30.3	2.27 (1H, m), 2.21 (1H, m)
3	26.2	1.23 (1H, m), 1.18 (1H, m)
4	26.9	1.84 (1H, m), 1.74 (1H, m)
5	35.9	1.23 (1H, m), 1.05 (1H, m)
6	33.6	1.26 (1H, m)
7	19.1	0.80 (3H, m)
8	28.9	1.26 (1H, m), 1.07 (1H, m)
9	11.2	0.80 (3H, m)
1-NH	----	8.10 (1H, t, 5.80)
10	42.1	3.72 (2H, m)
11	170.9	---
11-NH	----	8.13 (1H, d, 6.69)
12	52.1	4.27 (1H, m)
13	29.8	2.00 (1H, m), 1.87 (1H, m)
14	36.1	3.02 (1H, d, 8.52),2.86 (1H, d, 9.28)
14-NH_2		Not assigned
15	171.5	---
15-NH	---	8.06 (1H, m)
16	42.0	3.81 (1H, dd, 16.92, 5.92), 3.74 (1H, m)
17	168.9	---
17-NH	---	8.06 (1H, m)
18	50.9	4.35 (1H, d, 7.83)
19	61.4	3.60 (1H, m), 3.51 (1H, dd, 11.12, 5.73)
19-OH		Not assigned
20	171.2	---
20-NH	----	8.23 (1H, s)
21	53.9	4.52 (1H, dd, 13.46, 7.71)
22	27.3	3.04 (1H, d, 8.52), 3.20 (1H, m) (repeat)
23	109.8	----
24	123.8	7.18 (1H, d, 2.14)
24-NH	---	10.78 (1H, s)
25	136.0	----
26	118.4	7.58 (1H, d, 8.00)
27	120.8	7.05 (1H, t, 7.46)
28	118.2	6.97 (1H, t, 7.46)
29	111.3	7.32 (1H, d, 8.00)
30	127.3	----
31	171.4	----
31-NH	----	8.16 (1H, d, 6.69)
32	55.0	4.21 (1H, m)

33	61.6	3.70 (1H, m), 3.59 (1H, m)
33-OH		Not assigned
34	170.2	----
34-NH	----	8.06 (1H, m)
35	50.3	4.47 (1H, m)
36	30.5	1.98 (1H, m), 1.85 (1H, m)
37	36.0	2.84 (1H, t, 7.80), 2.75 (1H, t, 7.80)
37-NH_2		Not Assigned
38	170.	---
38-NH	----	8.24 (1H, d, 6.67)
39	55.1	4.29 (1H, m)
40	29.4	2.05 (1H, m), 1.94 (1H, m)
41	36.2	3.19 (1H, dq, 13.66, 3.42), 2.86 (1H, d, 9.28)
41-NH_2		Not assigned
42	170.6	----
42-NH	----	8.41 (1H, d, 6.67)
43	55.4	4.38 (1H, dd, 13.46, 7.77)
44	36.2	1.72 (1H, m)
45	15.2	0.84 (3H, m)
46	24.3	1.44 (1H, m), 1.12 (1H, m)
47	10.8	0.77 (3H, m)
48	169.5	----
48-NH	----	8.30 (1H, d, 7.71)
49	50.2	4.37 (1H, dd, 13.46, 7.77)
50	25.4	1.44 (2H, m)
51	35.2	2.11 (2H, t, 7.55)
52	172.9	----
53	170.8	----
53-NH	-----	8.39 (1H, d, 5.69)
54	54.9	4.26 (1H, m)
55	36.4	1.92 (1H, m)
56	14.5	0.82 (3H, m)
57	25.9	1.22 (1H, m), 1.10 (1H, m)
58	11.6	0.80 (3H, m)
59	171.7	----
59-NH	----	7.88 (1H, br s)
60	57.6	4.25 (1H, m)
61	36.9	1.74 (1H, m)
62	15.4	0.80 (3H, m)
63	24.0	1.28 (1H, m), 1.06 (1H, m)
64	11.0	0.79 (3H, m)
65	171.9	----
65-NH	----	8.17 (1H, d, 6.69)
66	56.5	4.23 (1H, m)
67	59.2	3.51 (2H, m)
67-OH	----	Not assigned

68	170.7	----

Chemical Formula: $C_{68}H_{113}N_{17}O_{20}$ Exact Mass: 1487.8348

*Some assignments may be switched due to overlap

Table-S2 Amino-acid specificity of different adenylation domains present in tridecaptin M biosynthetic gene cluster

Domain	Monomer specificity					
	Software predicted					Experimental
	Stachelhaus code	NRPSPredictor3 SVM	pHMM	PrediCAT	SANDPUMA ensemble	
1	Ile/val	Val	Val	N/A	Val	Gly
2	Dab	Orn	Dab	Dab	Ala	Dab
3	Ser	Ser	Ser	Ser	Dab	Gly
4	Gly	Gly	Gly	N/A	Gly	Ser
5	Trp	Phe	Trp	N/A	Trp	Trp
6	Ser	Ser	Ser	Ser	Dab	Ser
7	Dab	Orn	Dab	Dab	Ala	Dab
8	Dab	Orn	Dab	Dab	Ala	Dab
9	Phe	Phe	Phe	N/A	Phe	Ile
10	Glu	Glu	Glu	N/A	Glu	Glu
11	Ile/aIle	Ile	Val	N/A	Ile	Ile
12	Ile/aIle	Ile	Val	N/A	Ile	Ile
13	Ser	Ser	Ser	Ser	Dab	Ser

Supplementary figures

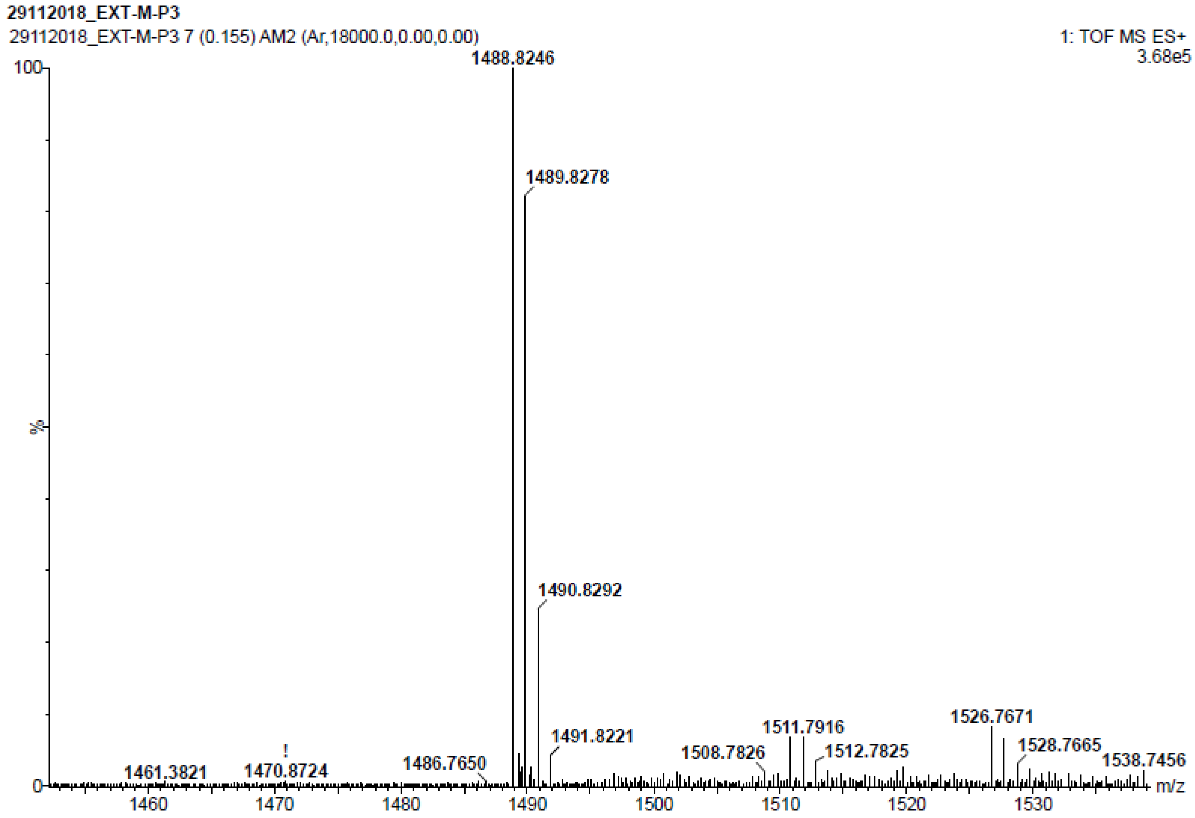

Figure S1. HR-ESI-MS of M152-P3 showing an m/z of 1488.8246 $[M+H]^+$

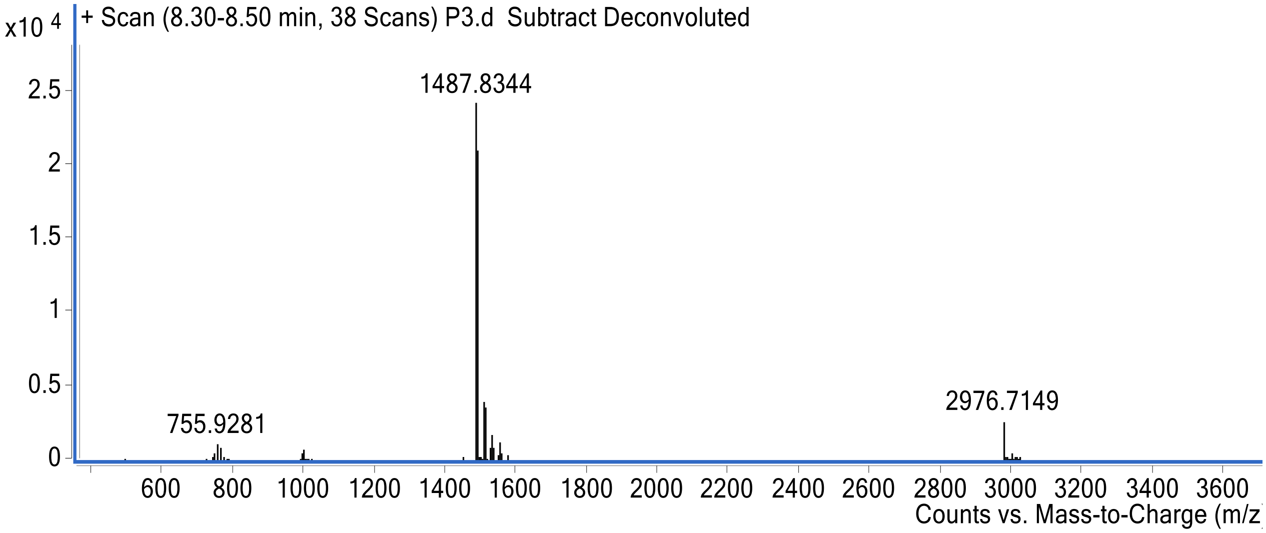

Figure S2. LC-ESI-MS of M152-P3. Monoisotopic mass is shown after deconvolution.

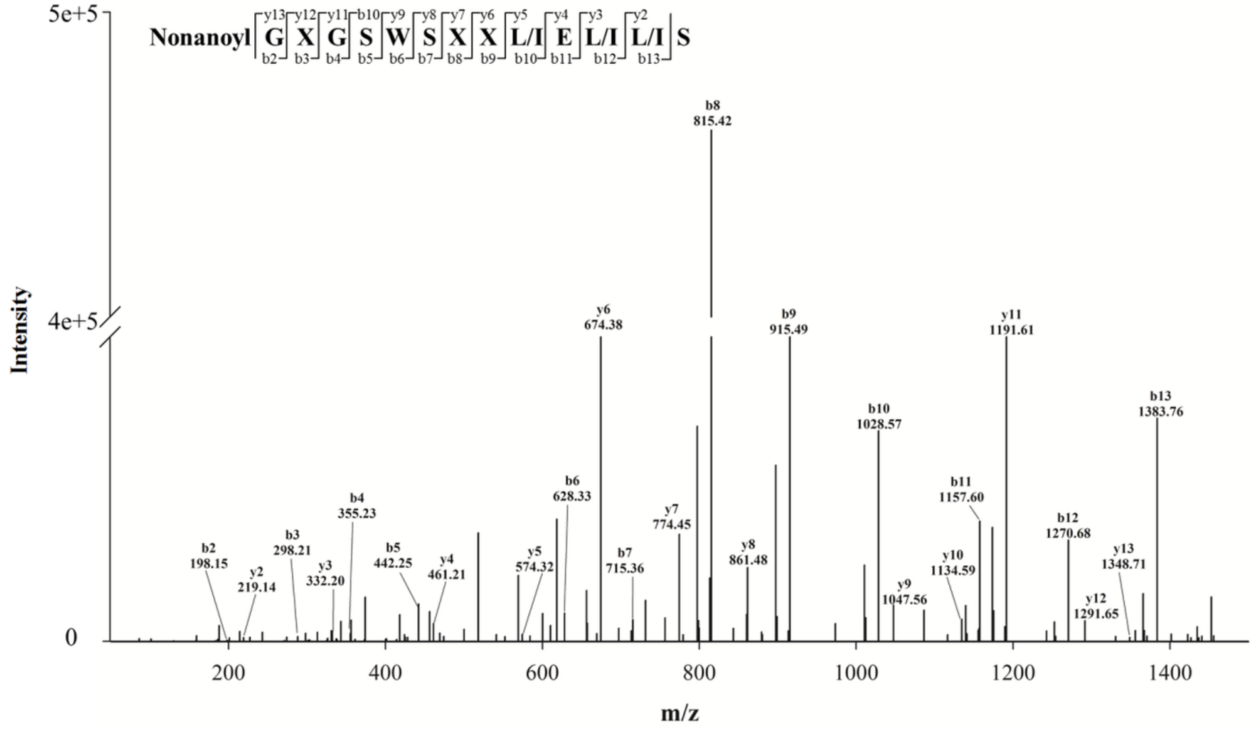

Figure S3. The figure represents MS/MS sequence of the compound M152-P3 where X is 2,4-diaminobutyric acid (Dab). At amino acid position 9, 10 and 11, either leucine or isoleucine is present because these two amino acids are undistinguishable in MS/MS spectrum due to similar masses. Since tridecaptin family contains an acyl moiety at its N-terminus, we proposed the presence of nonanoic acid (MW: 158 Da according to mass spectrum). Whether it is linear fatty acid or branched needs to be confirmed further and could not be determined with MS data. NMR spectroscopy later confirmed it as 6-methyloctanoic acid as explained in main text.

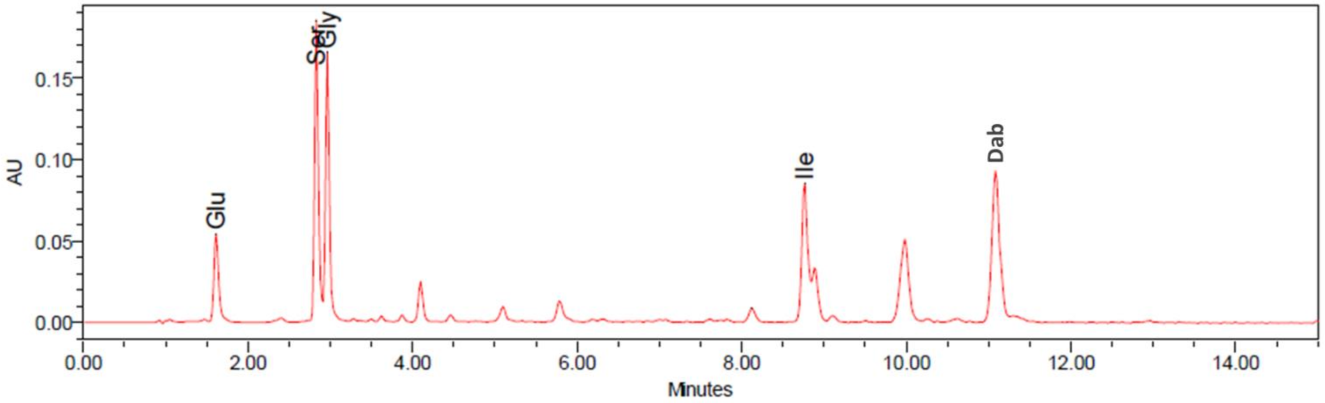

Figure S4. Amino acid analysis of M152-P3. The unknown peak at RT 10 min may be some impurity since it was also present in the chromatogram of standard amino acids mixture.

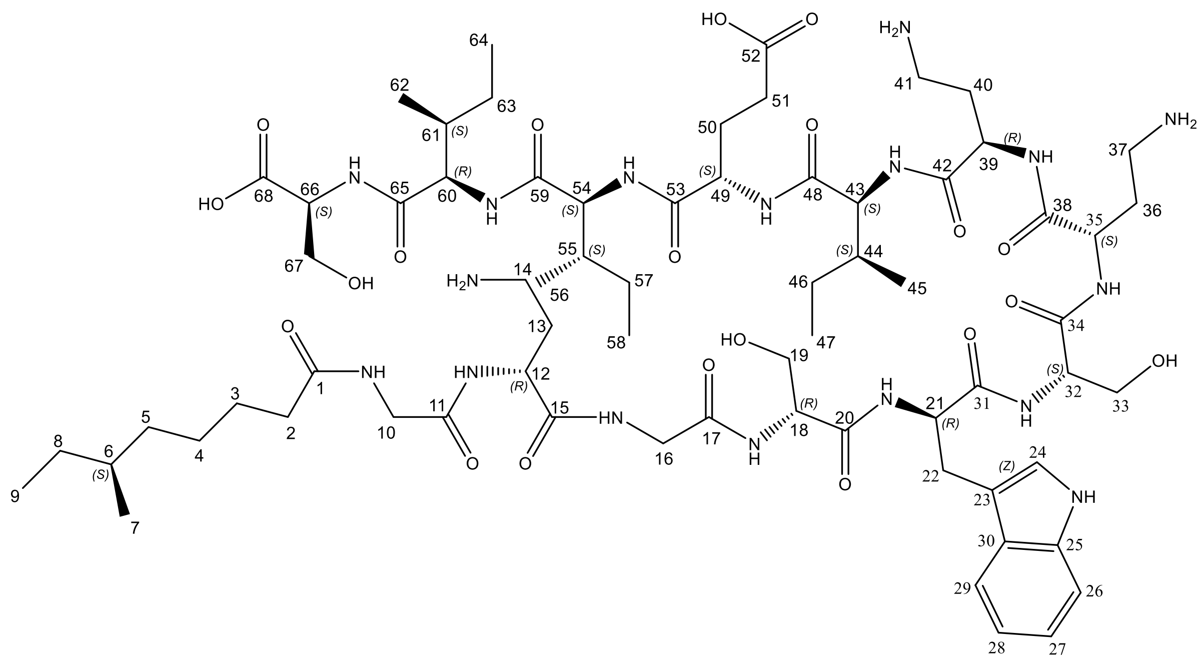

Figure S5. Structure of Tridecaptin M (M152-P3)

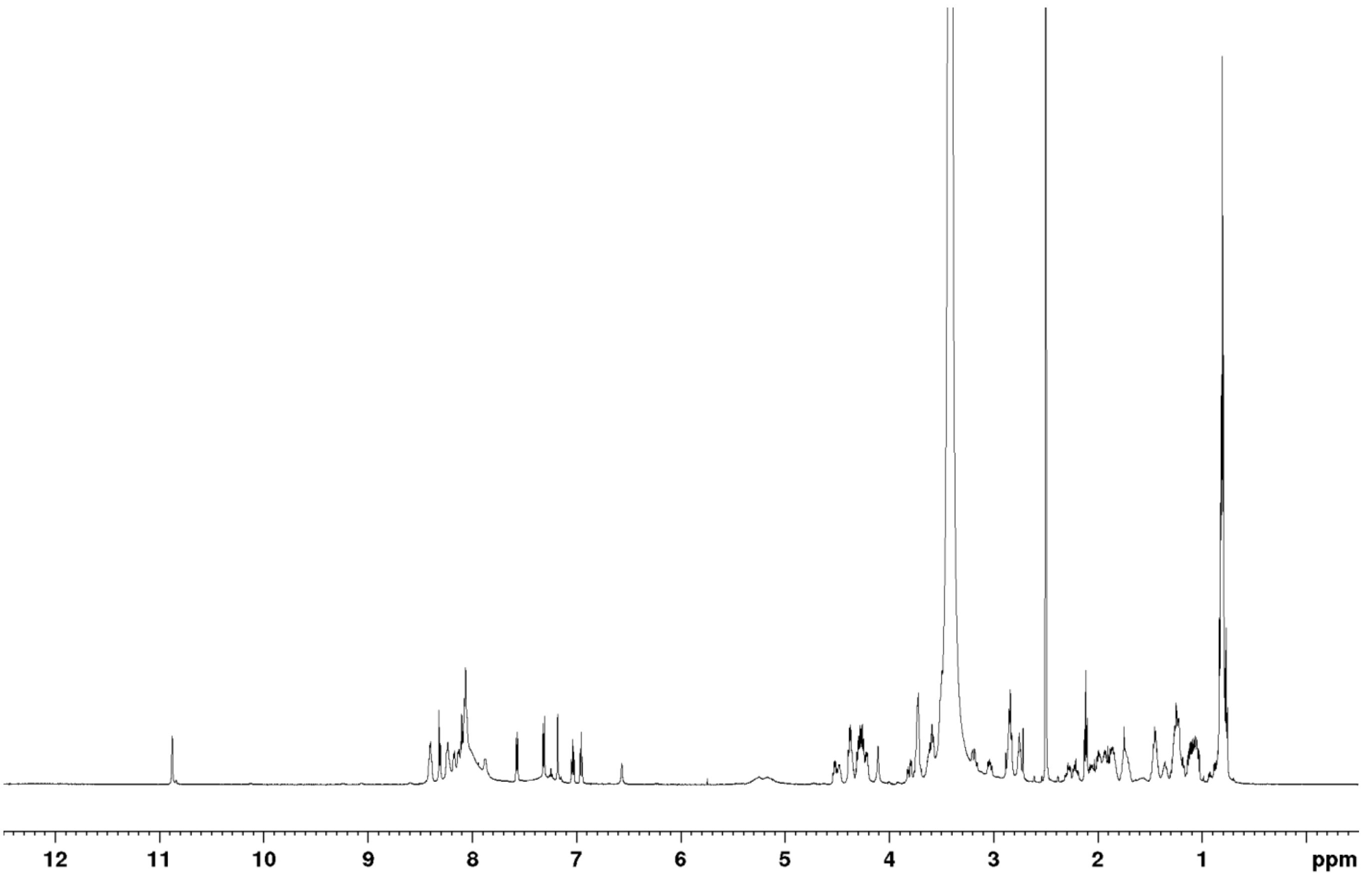

Figure S6. ^{1}H Spectrum (DMSO-d_6, 600 MHz)

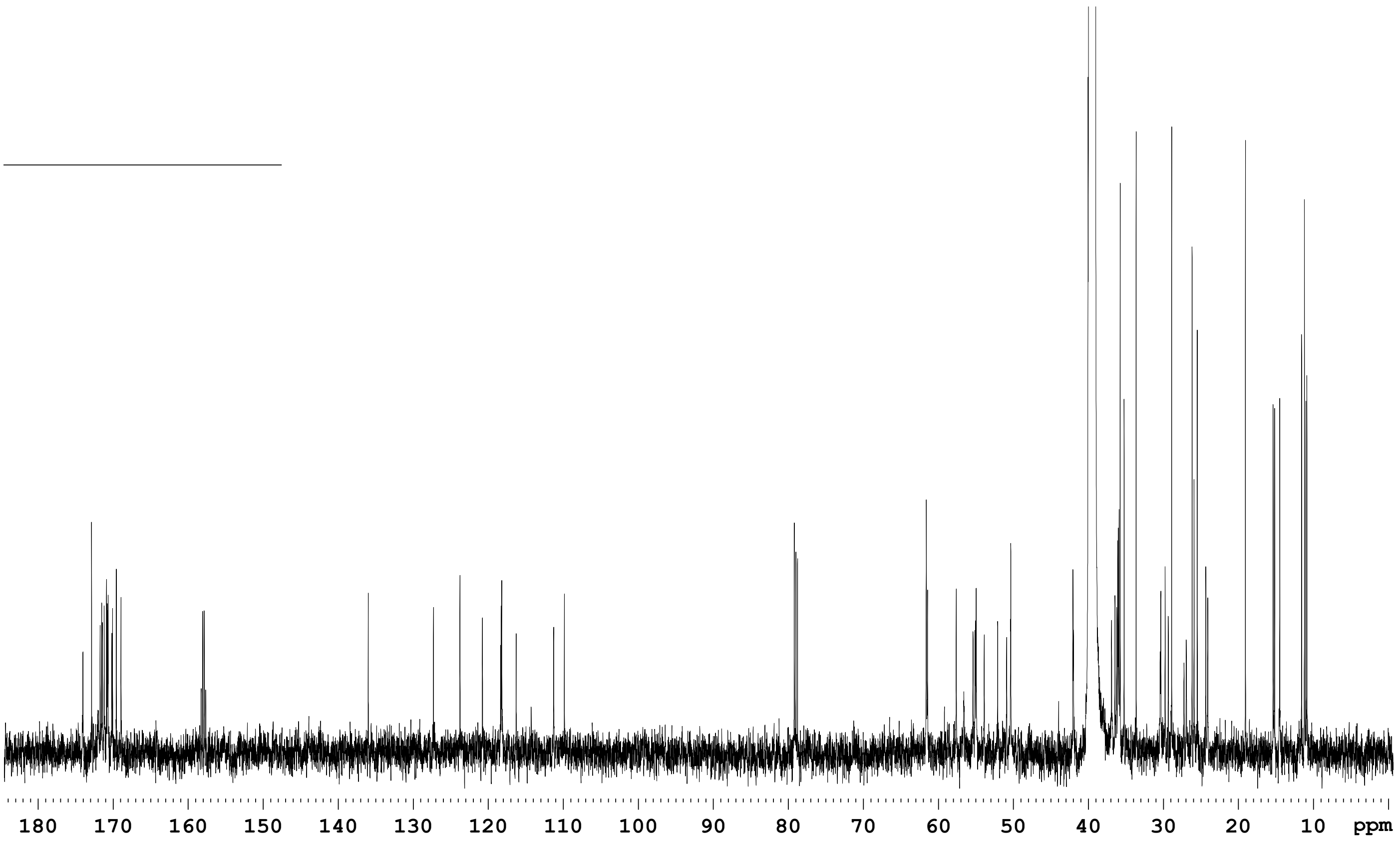

Figure S7. ^{13}C Spectrum (DMSO-d_6, 151 MHz)

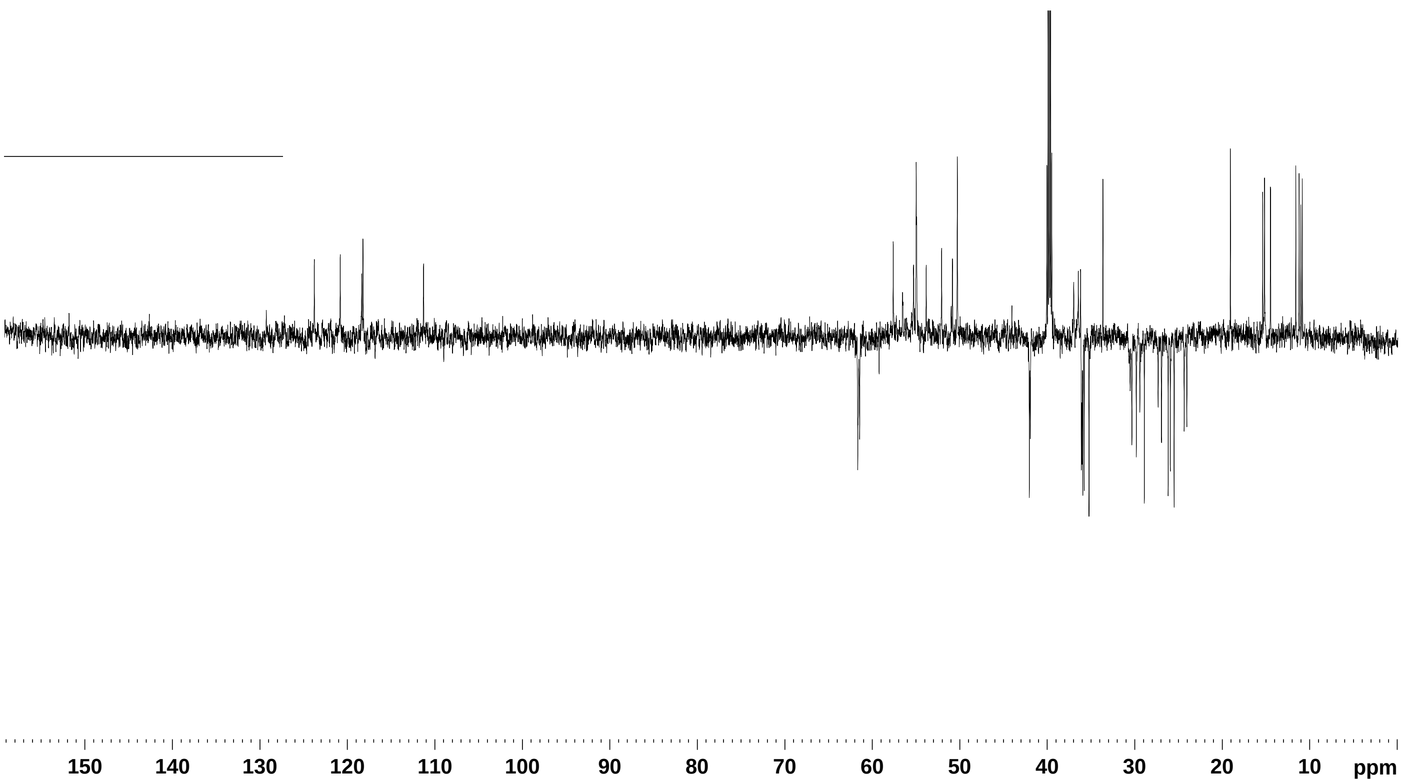

Figure S8. ^{13}C DEPT-135 Spectra (DMSO-d_6, 151 MHz)

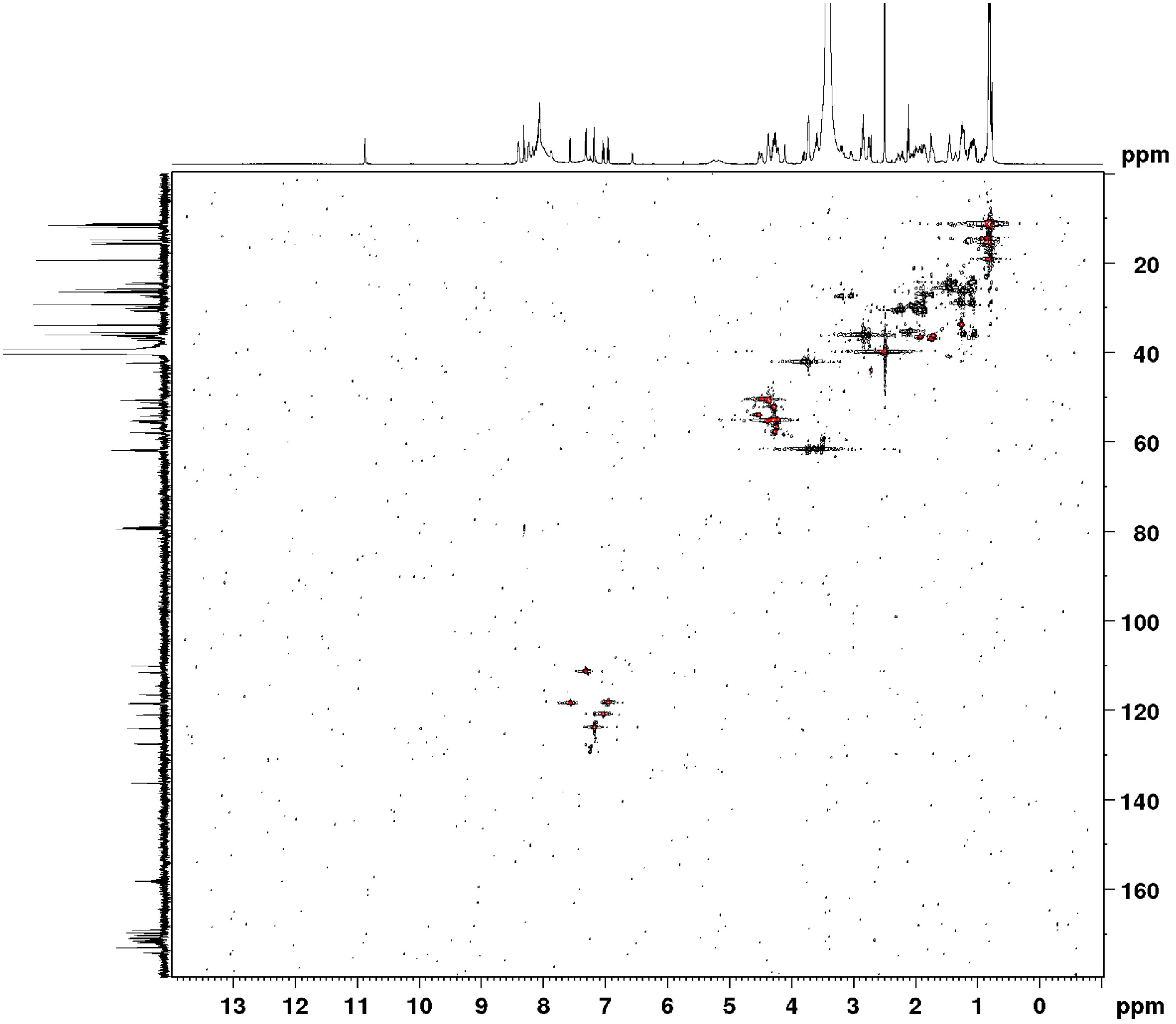

Figure S9. ^{1}H-^{13}C HSQC spectrum

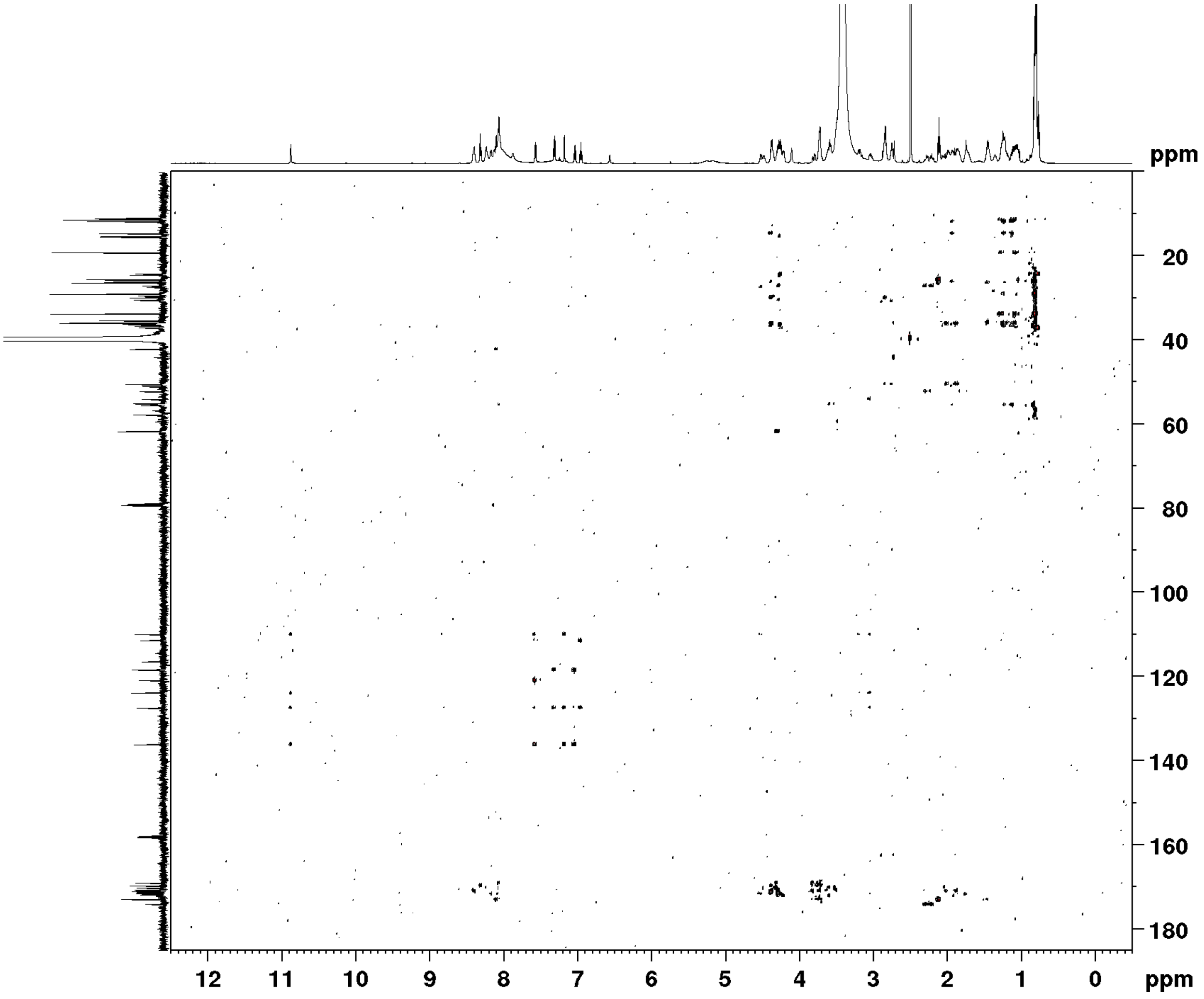

Figure S10. HMBC spectrum

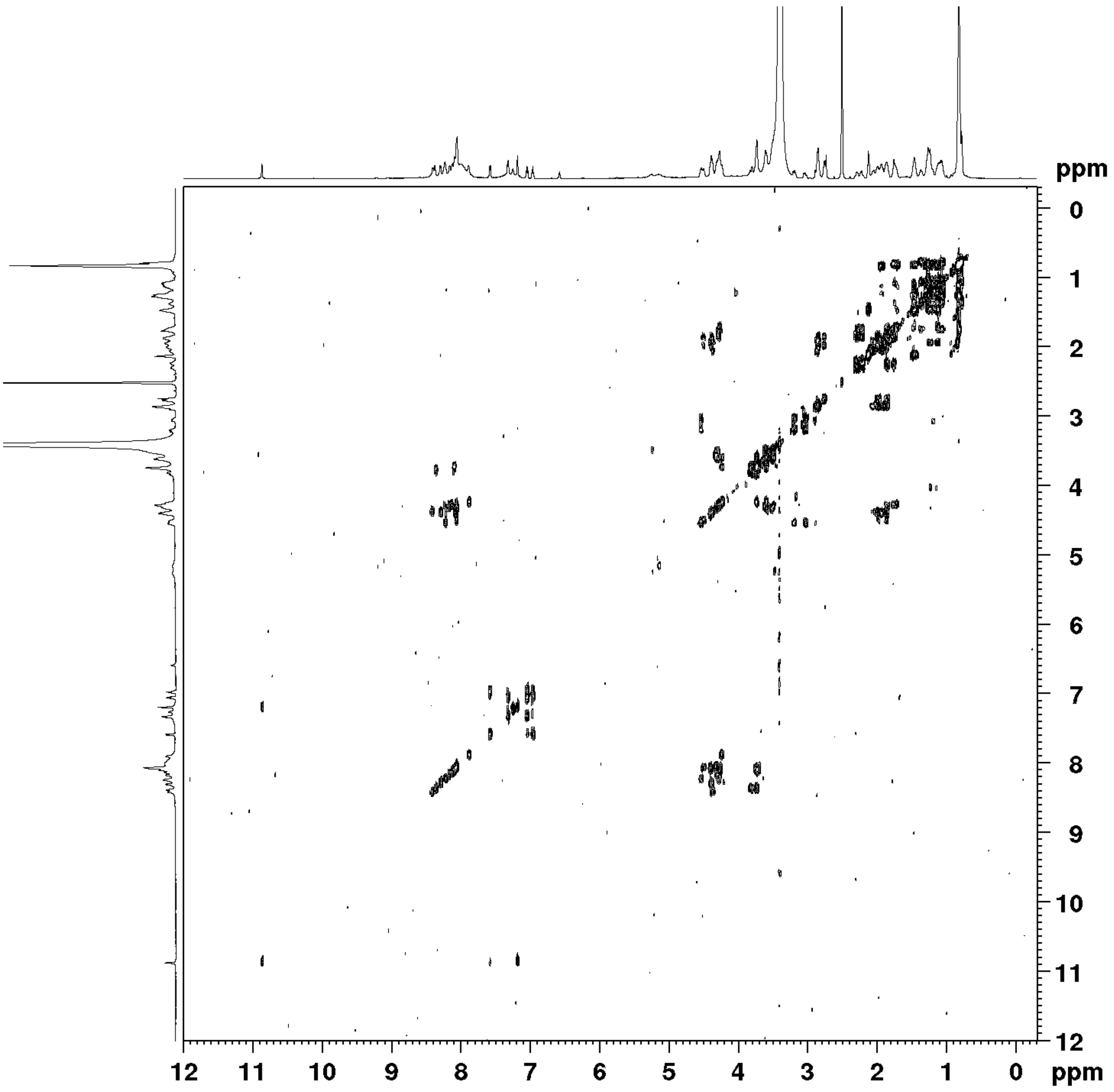

Figure S11. ^{1}H-^{1}H COSY spectrum

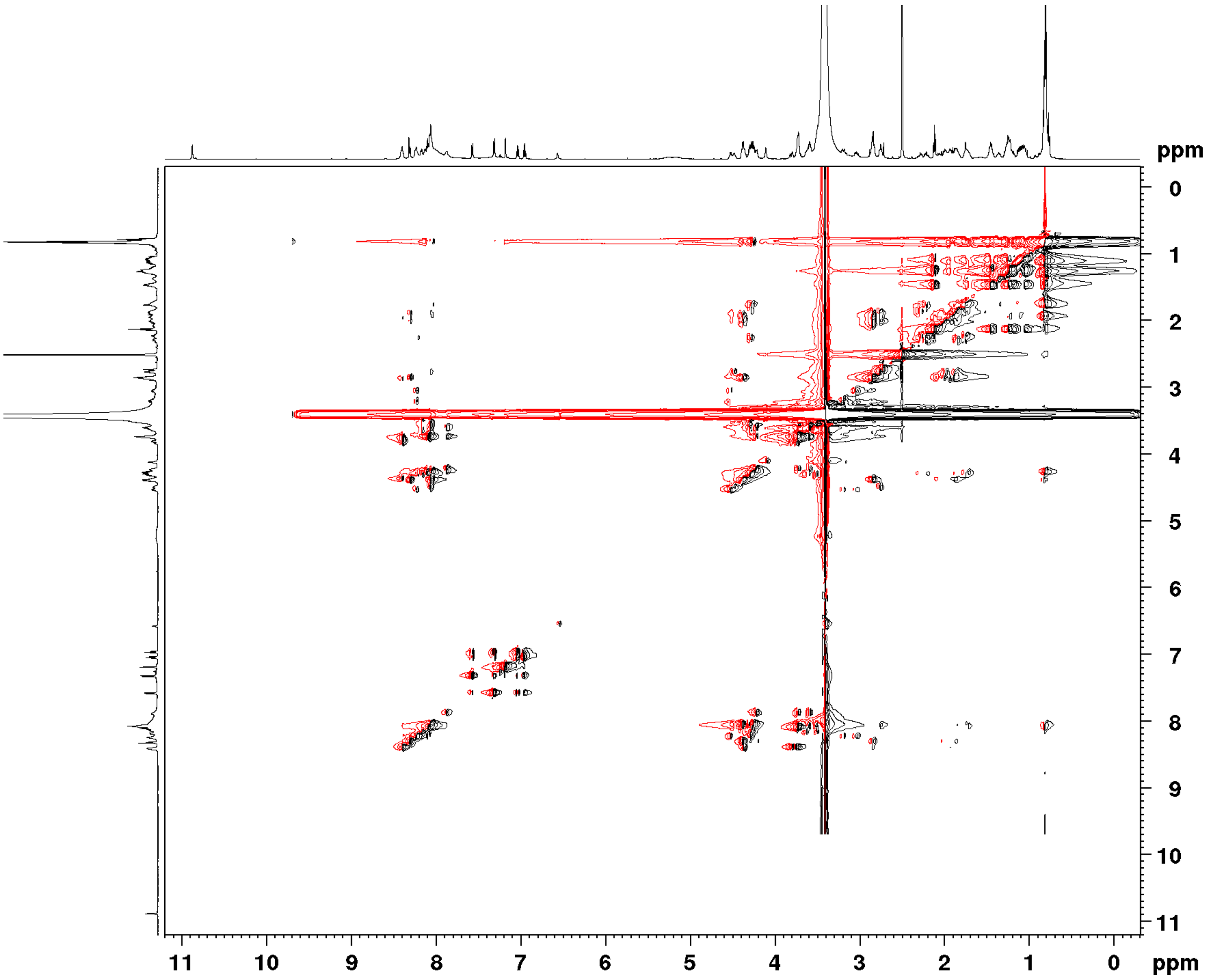

Figure S12. TOCSY spectrum
